# Yield Prediction of Organic Reactions in Biased Data Sets via Positive-Unlabeled Learning

**DOI:** 10.1021/jacs.6c00127

**Published:** 2026-04-01

**Authors:** Florian Boser, Jan C. Spies, Frank Glorius

**Affiliations:** 9185 Organisch-Chemisches-Institut, Universität Münster, Corrensstraße 36, 48149 Münster, Germany

## Abstract

The vast reaction data within scientific literature represents a rich resource for training predictive machine learning models. However, this resource is fundamentally compromised by a pervasive selection and reporting bias, resulting in imbalanced data sets. In this work, we introduce “Positivity is All You Need” (PAYN), a machine learning framework that addresses this data-scarcity problem by learning directly from biased, positive-only data. PAYN leverages a spy-based positive-unlabeled (PU) learning strategy, treating reported high-yielding reactions as the “positive” class and the vast, unexplored chemical space as the “unlabeled” class. To validate our approach, we simulated literature bias on fully labeled high-throughput experimentation (HTE) data sets, including Ni-catalyzed borylations, Buchwald–Hartwig and Suzuki–Miyaura couplings. We demonstrated that PAYN significantly improves the performance of models trained on biased data by balancing the data with augmented negative data points. This work establishes a robust strategy for leveraging biased data, paving a path toward more scalable and accessible data-driven strategies for accelerating synthesis design, optimization, and chemical discovery.

## Graphical abstract

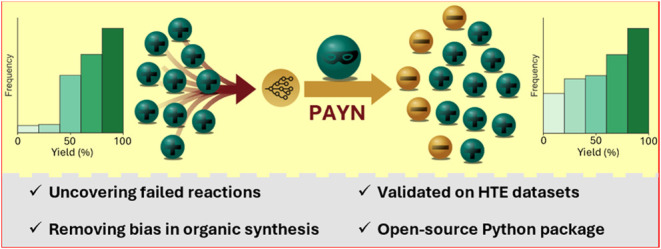

## Introduction

The accurate *a priori* prediction of reaction yields represents a long-standing objective in organic chemistry.^1^ Achieving this goal would revolutionize chemical synthesis, enabling chemists and automated platforms to streamline reaction discovery, accelerate reaction optimization, and rationally guide the design of complex retrosynthetic routes, thereby enhancing overall efficiency and sustainability.^2–9^ The impact would be particularly profound in medicinal chemistry and high-throughput experimentation (HTE), where predictive models could facilitate the construction of synthetically accessible virtual libraries and maximize the efficiency of experimental designs.^10–13^ To this end, the vast collection of reaction data accumulated in the scientific literature and patent databases offers an unprecedented resource for developing predictive machine learning (ML) models that have been already successfully employed for retrosynthesis prediction in the past.^14–17^ This resource spans several decades and encompasses an extraordinary diversity of chemical transformations.^18–21^

However, the utility of this data for yield prediction is severely constrained by two pervasive biases.^22^ First, a selection bias emerges from the tendency of chemists to favor familiar conditions, established protocols, and commercially accessible reagents—a rational strategy to ensure a high likelihood of success that, however, consequently limits exploration of the wider chemical space (Figure 1B).^23,24^ Second, the yield distribution is distorted by a reporting bias: even when diverse experiments are conducted, failed or low-yielding reactions remain systematically underreported (Figure 1C).^25–28^ This ongoing practice creates highly skewed data sets, rich in “positive ”examples (high-yielding reactions) but critically deficient in “negative” examples (low yielding or unproductive reactions), which are essential for training predictive models that can distinguish success from failure (Figure 2A).^29,30^ The consequences of this imbalance are profound: supervised ML approaches trained on such data sets tend to overestimate yields and fail to generalize, particularly when tasked with identifying unproductive reactions—the very capability required to save resources in the laboratory.^31–33^ Intriguingly, recent work by Gao et al. has attempted to reframe this problem by treating the bias itself as a source of information, learning chemical reactivity patterns through contrastive learning from the coreporting of substrates in the literature.^34^

**Figure 1:**
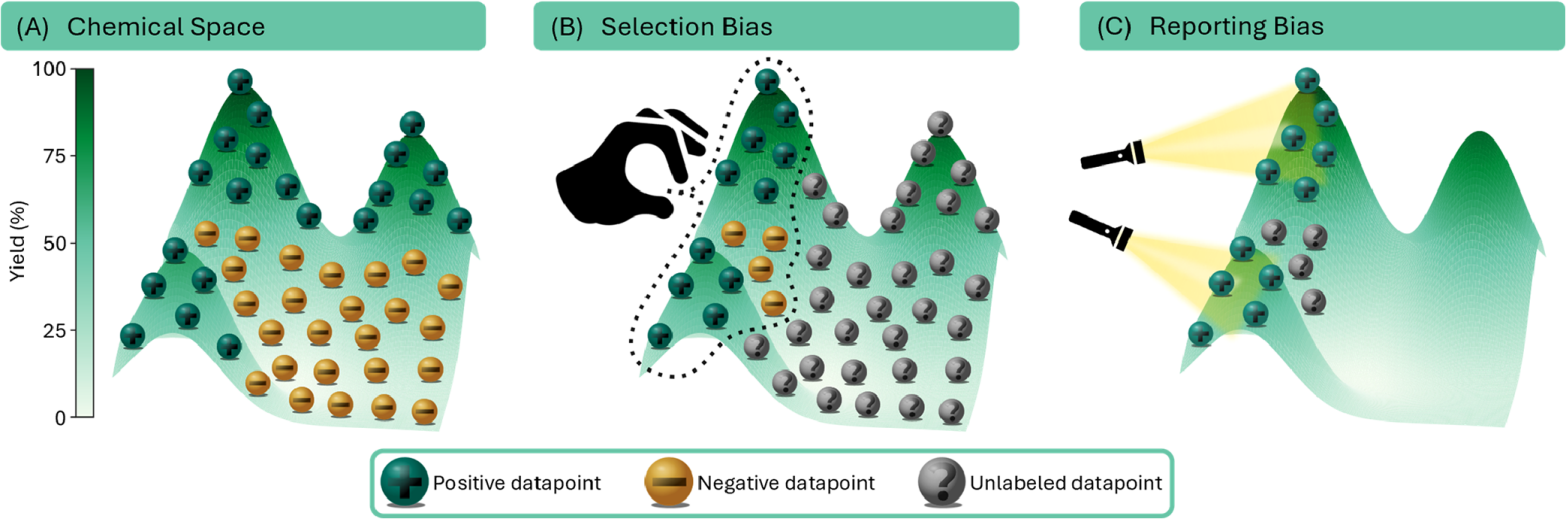
(A) The true chemical space contains a vast number of successful (high-yielding, green) and unsuccessful (low-yielding or failed, gold) reactions. (B) Selection bias causes chemists to preferentially explore familiar and established regions of this space, leading to a high number of untested/unlabeled experiments (gray). (C) Reporting bias leads to the overwhelming publication of successful outcomes, while unsuccessful experiments are rarely reported.

**Figure 2:**
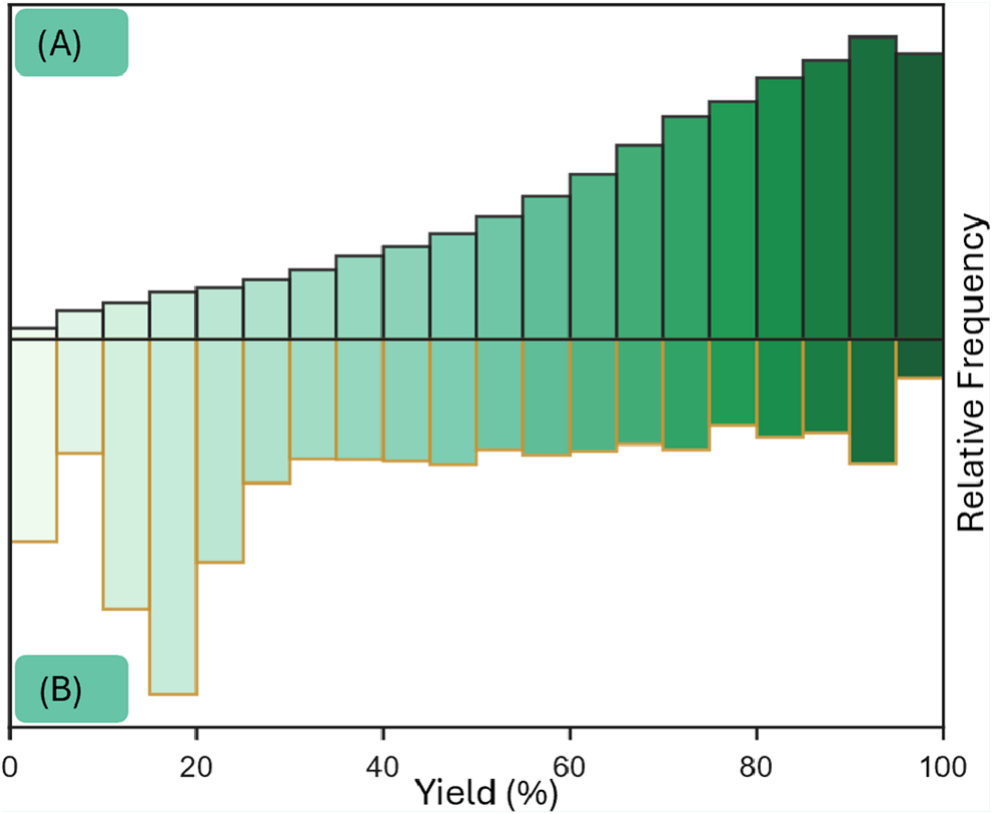
Comparison of binned yield frequencies for Suzuki–Miyaura couplings. (A) Literature databases such as Reaxys are severely skewed toward high-yielding examples.^20^ (B) HTE Data set by Perera et al. shows a more balanced yield distribution.^38^

In response to these biased data sets, the field has increasingly turned toward HTE to systematically perform a large number of reactions under standardized conditions. (Figure 2B).^35–42^ HTE-derived data sets, featuring both positive and negative outcomes, have thus enabled the construction of more generalizable yield predictive models that more faithfully capture the contours of the true reaction landscape (Figure 1A).^2,9,43–46^ However, yield predictive models developed on the basis of HTE data typically exhibit strong performance only within their narrow application domains defined by the underlying data, and often exhibit limited generalizability to broader chemical contexts.^27,37,38,43^ Moreover, individual HTE campaigns are in turn constrained by considerable investment in dedicated HTE centers or substantial investment in robotic infrastructure, dedicated analytics, consumables, and chemicals—constraints that remain prohibitive for the majority of research groups.^29,40,47^

Therefore, the broad and diverse chemical landscape and knowledge contained in the scientific literature and patent databases will be impossible to reproduce in HTE in the foreseeable future. Consequently, there is a critical imperative to devise computational strategies that can reconstruct the missing negative counterpart to the vast historical record of successful chemistry, rendering this unexploited resource accessible for modern artificial intelligence.

In this work, we introduce “Positivity is All You Need” (PAYN), an ML Python framework for the robust extraction and identification of negative data points from biased data. PAYN leverages positive-unlabeled (PU) learning to compensate for the absence of negative data by making use of an unlabeled data set. The unlabeled data set consists of unreported or unexplored reactions, a mixture containing both true negative data points and yet undiscovered positives.^48,49^

Known positive (successful reactions) examples are then used as a guide to systematically identify and extract reliable negatives from the unlabeled set. This paradigm has proven effective in fields ranging from materials discovery^50–53^ and bioinformatics^54–61^ to text classification.^62–65^

We demonstrate and benchmark our PU approach utilizing fully labeled HTE data sets as a ground truth. We have previously demonstrated that reporting bias is the most detrimental to the prediction error of yield predictive models.^25^ By masking known negative outcomes, we simulated the reporting bias inherent in the literature data. Within multiple case studies covering three different reaction types, PAYN demonstrates an unprecedented ability to identify low-yielding reactions, indicating a potential path toward unlocking the vast chemical literature for predictive modeling. During the finalization of our studies, Nishii et al. applied PU learning as a reactivity informer in the oxidative homocoupling of phenols.^66^ We expect further uptake of PU learning in the community and hope to contribute with our open-source Python framework.

## Positive-Unlabeled Learning

To understand how PAYN extracts valuable information from biased data, PU learning must first be formalized. In a supervised binary classification (positive–negative learning), a model differentiates between two labeled sets: positive examples (*y* = 1, e.g., successful reactions) and negative examples (*y* = 0, e.g., failed reactions). Since reported chemical reactions are mostly positive, we lack the negative counterparts (Figure 2A). Instead, we face a vast unlabeled space, consisting of the unexplored chemical landscape. Crucially, this unlabeled space is a mixture of two hidden classes: 1. True negatives (*y* = 0): unsuccessful or low-yielding reactions. 2. Latent positives (*y* = 1): reactions that would succeed but have not yet been discovered or reported. Formally, we do not observe the true label *y*, but rather a label variable *s*, where *s* = 1 if a reaction is reported, and *s* = 0 if it is unlabeled. To mathematically fund PU, a few assumptions must be made, including the “selected completely at random” (SCAR) assumption.

This posits that the reported examples (*s* = 1) are a representative, independent sample of the true positive distribution (*y* = 1), regardless of their specific features *x*. This SCAR assumption formulated by Elkan et al. implies that the probability of a reaction being reported depends only on whether it works (*y* = 1) and a constant labeling probability *c* = *p*(*s* = 1|*y* = 1):^67^

p(s=1|x,y=1)=p(s=1|y=1)

This leads to the central insight into PU learning: although a standard binary classifier trained on PU data predicts the probability of a reaction being reported (*s* = 1), this prediction is directly proportional to the true probability of the reaction being successful (*y* = 1):^67,68^

p(s=1|x)=p(y=1|x)c

Consequently, a model trained on PU data correctly ranks molecular features: a reaction with a higher predicted score is chemically more likely to work, enabling the discrimination of latent positives from true negatives within an unlabeled set. Further premises for PU learning are separability—the positive and negative reactions must be distinguishable in the feature space—and smoothness—that reactions with similar features are likely to have similar outcomes.^67^

### Identification of Reliable Negatives via Spy Technique

PAYN is specifically designed to extract “reliable negatives” to balance biased data sets. To achieve this without prior knowledge of the negative class, we employ a spy technique (Figure 3), originally introduced by Liu et al.:^69^

1. Infiltration: A random sample of our known positive reactions is selected as spies and injected into the unlabeled set, with their labels concealed (*s* = 0, Figure 3A,B).
2. Training: A binary classifier is trained to distinguish the remaining known positives from the unlabeled set, now containing unknown negatives, unknown positives, and the spies (Figure 3C).
3. Probability prediction: Leveraging the smoothness and separability assumptions, the model will assign relatively high probability scores to the spies, despite them being labeled as negative during the training (Figure 3D).
4. Thresholding: By examining the probabilities assigned to the spies, a new threshold *t*_spies_ can be established, separating the majority of spies from unlabeled data points with lower probabilities (Figure 3E).

**Figure 3:**
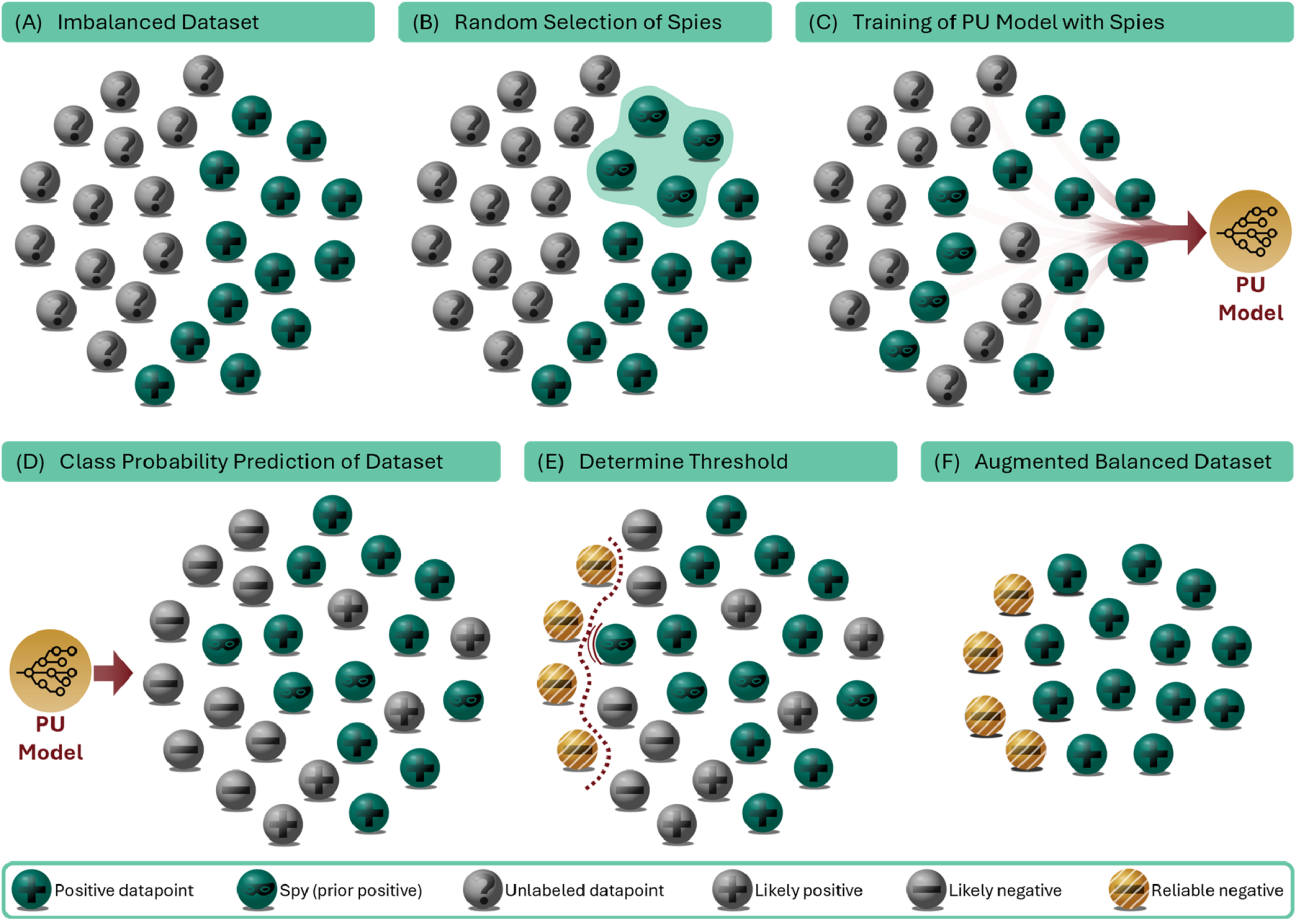
Process of spy-based PU learning. (A) The starting point of PU learning is an initial biased data set, comprising known positive data points and a pool of unlabeled data points. (B) A small, random subset of the positive data is selected as spies and injected into the unlabeled set with the true labels concealed. (C) A binary PU classifier is trained to distinguish the remaining positive examples from the spy-containing unlabeled set. (D) The class probability of the trained model is computed for each unlabeled data point. (E) A threshold separating reliable negatives from latent positive data points is calculated according to class probability predictions of the spies. (F) Combination of found reliable negatives and known positive results in a balanced data set.

Any unlabeled data point *x*_u_ is classified according to the predicted probability *p*(*s* = 1|*x*_u_). If this probability is below the spy-derived threshold *t*_spies_, the data point is considered a reliable negative (RN):

$$\mathrm{RN}=\left\{x\in U|p\left(s=1|x\right)\leq t_{\mathrm{spies}}\right\}$$

In combination with the originally known positives, this allows the generation of an augmented, more balanced training set (Figure 3F).

## Results and Discussion

### Simulation of Reporting Bias

To establish a controlled environment for benchmarking our method, we utilized three distinct HTE data sets covering various regions of chemical space (Figure 4). The first data set by Ahneman et al. contains 3955 Buchwald–Hartwig couplings, the second by Stevens et al. contains 779 Ni-catalyzed borylation reactions, and the last by Perera et al. contains 5760 Suzuki–Miyaura couplings (Figure 5).^37,38,42^ Lastly, we applied our findings to the very recent noncombinatorial Buchwald–Hartwig data set from Neves et al.^46^

**Figure 4:**
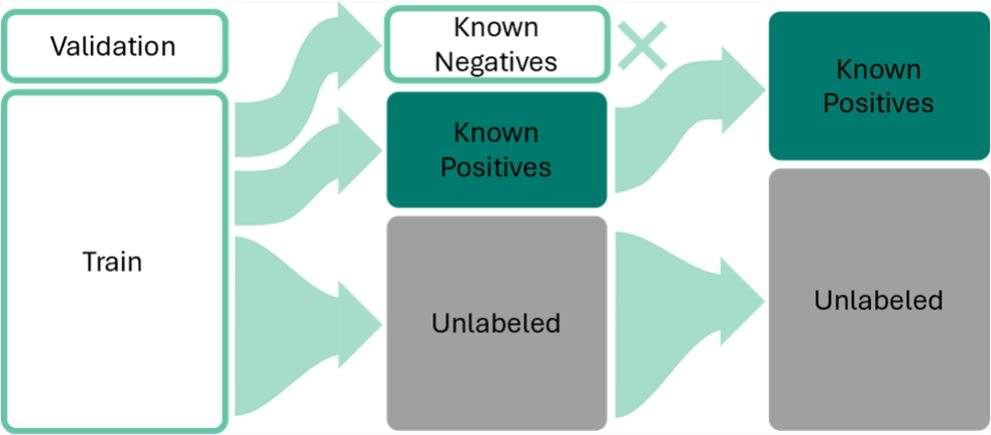
Fully labeled HTE data set is transformed into a positive-unlabeled data set. The unlabeled data contain the natural distribution of positive and negative data points with their true label concealed. The remaining data points are split into known negatives and known positive data points, with the negative data points being disregarded to simulate reporting bias.

**Figure 5:**
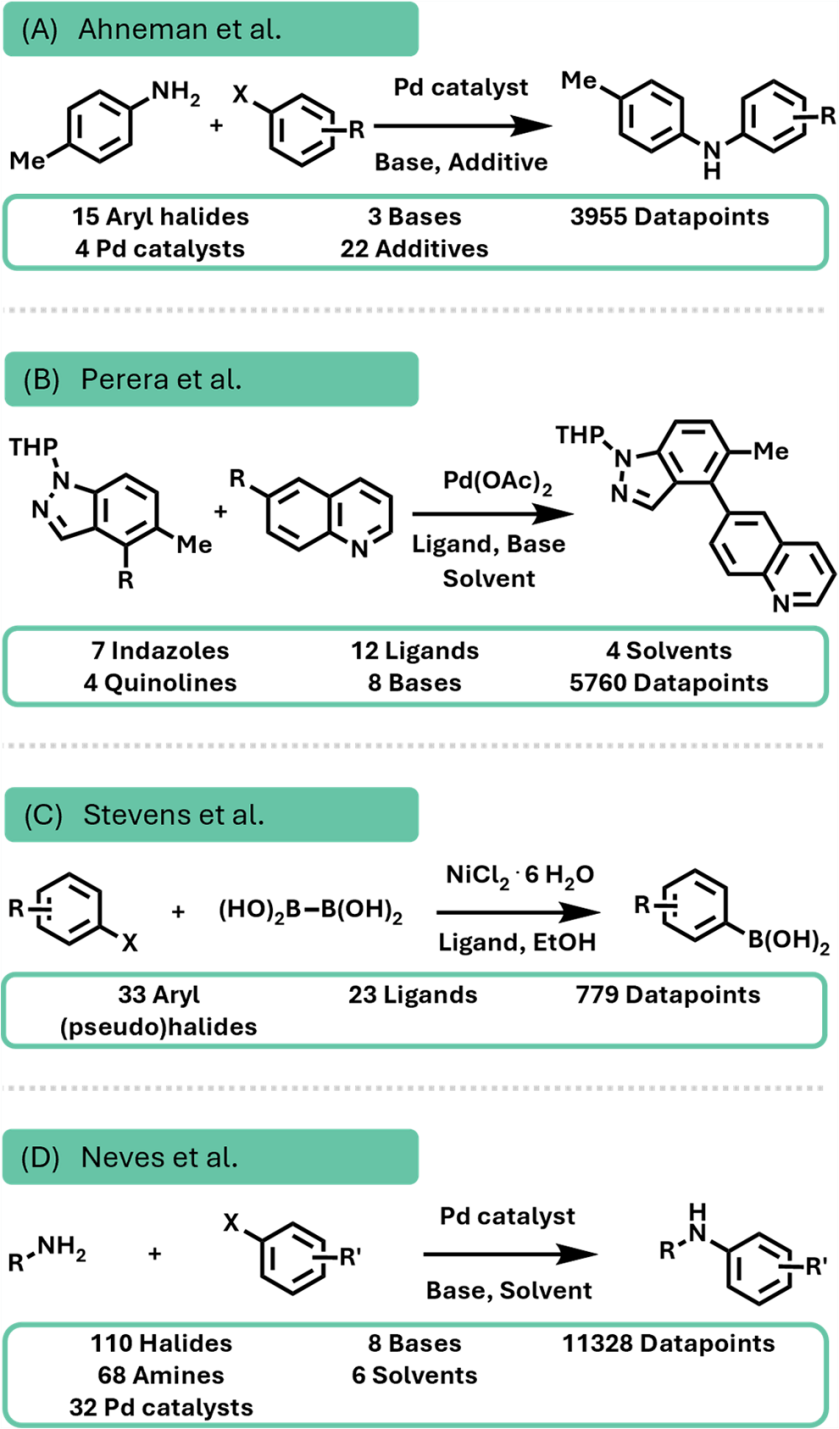
General reaction schemes of the HTE data sets used within this work to benchmark the PAYN framework. (A) Buchwald–Hartwig data set by Ahneman et al.,^37^ (B) Suzuki–Miyaura data set by Perera et al.,^38^ (C) Borylation data set by Stevens et al.,^42^ and (D) Buchwald–Hartwig data set by Neves et al.^46^

As the reaction yield is continuous (0–100%), we first framed the PU prediction task as a binary classification problem. A reaction was classified as positive (*y* = 1) if its yield exceeded 20%. We employed a 5-fold cross-validation using 10% of the training data as a validation set for hyperparameter optimization. Crucially, we then transformed the fully labeled training set into a PU data set to simulate reporting bias (Figure 4). A defined portion (controlled by the hyperparameter PU ratio) was stripped of negative data points to create the known positives. The remaining data points, comprising the rest of the true negatives and true positives, were pooled into an unlabeled set, with their true labels concealed (*s* = 0). This procedure directly mimics the data-scarcity problem in the literature through reporting bias, where only positive reactions are reported (*s* = 1), and the status of the remaining chemical space is unknown (*s* = 0). While our simulation explicitly models reporting bias, HTE data sets, and literature data in general, are also shaped by selection bias. Substrates and conditions are selected based on chemical intuition, precedent, and established reactivity rules, specifically to maximize the probability of success. We hypothesize that the intrinsic sparsity of successful reactions in the global chemical space makes chemistry particularly amenable to PU learning. In a truly random selection of substrates, the probability of a successful reaction, *p*(*y* = 1), is extremely low (for details, see Supporting Information (SI), Section 3):

$$p\left(y=1|x\in U_{\mathrm{Selection Bias}}\right)\gg p\left(y=1|x\in U_{\mathrm{random}}\right)$$

### PAYN Framework

With the aim of turning the theoretical principles of PU learning into a practical tool for chemical discovery, we developed PAYN as a modular, open-source Python framework. The architecture allows tabular reaction data to be used seamlessly and automates the entire pipeline, including feature generation, data splitting, spy generation, and model training including Bayesian Optimization and evaluation. For the probabilistic base classifier, we employed CatBoost, a gradient-boosting algorithm chosen for its robust performance and speed of training.^70,71^ However, the framework is not limited to Catboost and the base model is interchangeable and adaptable, as long as a class probability prediction of the unlabeled data points can be generated or estimated.^72^ Bayesian Optimization of models is guided by Optuna, which was used for optimizing depth, learning rate, and iterations of CatBoost models.^73^ To ensure the framework’s adaptability across diverse chemical domains, we implemented the extended-connectivity fingerprints (ECFP) and multiple fingerprint features (MFF), but custom features such as precalculated density functional theory (DFT) descriptors are also supported.^9,74^ The entire workflow is controlled via a centralized configuration file, enabling the user to effortlessly modify data sets, settings, and hyperparameters. These hyperparameters include the spy-based learning process (SI, section 5 for further details):

1. PU ratio: Defines the experimental scenario by setting the degree of positive data point scarcity relative to the unlabeled space.
2. Spy rate: Defines the fraction of known positive data points that are masked and injected into the unlabeled set as spies. This parameter ensures that the model has a sufficient statistical sample of the latent positive distribution within the unlabeled set, relying on the smoothness assumption that spies and remaining positives share similar feature distributions.
3. Spy tolerance: Determines the probability threshold *t*_spies_ derived from the spy distribution. A tolerance of 5% sets the threshold such that 95% of the spies are correctly recognized as positive by the model. Unlabeled data points scoring below this threshold are classified as RN.

### Precise Identification of Reliable Negatives

The pivotal step in the PAYN pipeline is the extraction of RN to create a more balanced augmented data set. To evaluate the efficacy of this extraction process, we utilized a PU data set, created from the HTE data set by Ahneman et al. as a primary case study. For this analysis, all reactions were encoded using generic ECFP. While we acknowledge that sophisticated, domain-tailored descriptors could potentially enhance separability, the use of standard ECFP ensures that the framework’s performance is benchmarked against a universally accessible baseline, facilitating comparison across different chemical spaces. The efficacy of identifying RN hinges on a delicate trade-off between label fidelity and data set balancing. We posit that negative precision (percentage of RN that are true negatives) is crucial to this, as falsely flagging a latent positive as a negative introduces severe label noise, which degrades the resulting data set. At the same time, however, the negative recall (percentage of all negatives in the unlabeled set that are correctly identified) is important to balance the data set. For both negative precision and recall being high, a good separability of the underlying positive and negatives distribution of the unlabeled data by the PU classifier must be ensured. We therefore first assessed the classifier’s ability to distinguish between the latent positive and negative populations within the unlabeled space. The probability density distributions, visualized in Figure 6A, demonstrate that effective separation is achieved. Although there is some overlap, the two latent classes exhibit distinct profiles. Notably, the actual negatives (gold) are sharply concentrated in the low-probability region (<0.2), indicating that the classifier can differentiate these areas of chemical space from the labeled positives.

**Figure 6:**
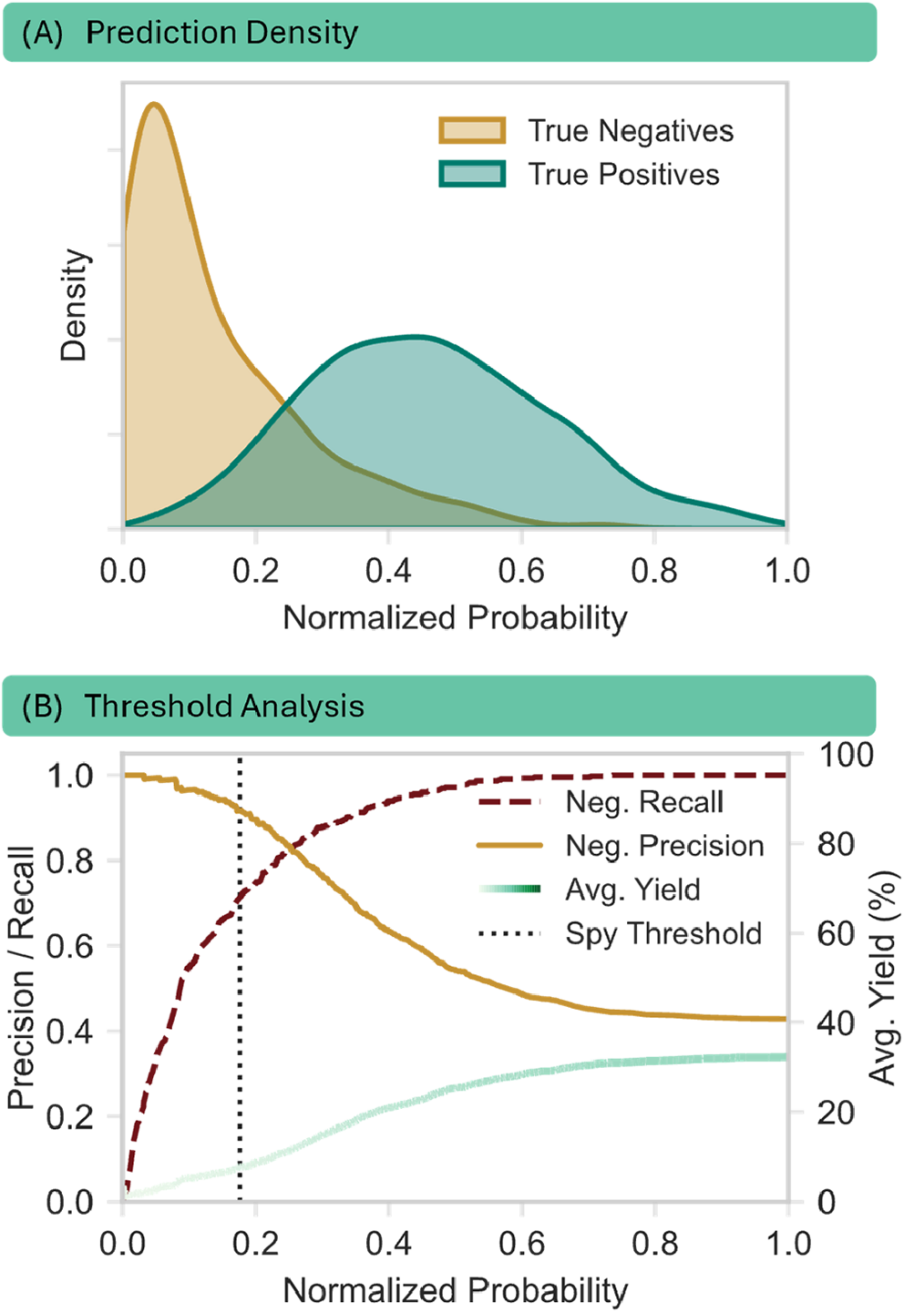
First fold of a model trained on the data set by Ahneman et al. is shown as an example. (A) Probability density distribution of the unlabeled data subset. The *x*-axis displays the normalized prediction scores assigned by the PU classifier. The distributions are stratified by their ground-truth labels: true negatives (gold) and true positives (green). The separation demonstrates the classifier’s ability to distinguish latent positives from negatives within the unlabeled pool. (B) The setting of a threshold can greatly influence the recall, precision, and average yield of the reliable negatives. PAYN dynamically picks the threshold via infusing spies into the unlabeled set. The thus determined threshold is shown in black.

In contrast, the latent positives (green) span a significantly broader probability space, exhibiting a high variance in their assigned scores (Figure 6A). We attribute this dispersion to the inherent ambiguity of the PU learning task: the classifier is trained to distinguish labeled positives from the unlabeled mixture. Because latent positives within the unlabeled set are chemically similar to the examples in the positive set, the model faces an objective conflict, forced to classify structurally analogous data points against each other. This results in greater epistemic uncertainty and a wider distribution of prediction scores for the latent positive class compared with the true negatives. Despite this variance, the bulk of the positive distribution remains sufficiently separated from the negative peak, allowing for the strategic placement of a classification threshold.

In general, determining the optimal cutoff to separate these overlapping distributions is nontrivial, especially in less well-separated cases. An arbitrarily high threshold ensures high recall but compromises the purity of the negative set, while an overly conservative threshold discards valuable negative data. Worse, between different data sets and even different folds, the distributions may vary, and the selection of a threshold must be adjusted to that. To automate this selection, the PAYN framework utilizes the “spy” technique. By injecting a subset of known positives into the unlabeled set, we can empirically model the behavior of latent positives and, thus, dynamically determine a threshold that excludes most of the latent positives from the RN.

Figure 6B quantifies this trade-off. As the classification probability threshold increases, the negative recall (red dashed line) rises but the negative precision (gold line) degrades. Crucially, we monitored the average yield of the predicted RN (green line) as a secondary validation metric. At low probability thresholds (<0.1), the average yield of the selected subset remains near zero, confirming that the model is successfully isolating nonfunctioning reactions. However, as the threshold enters the region of ambiguity (approximately 0.2–0.4), the average yield begins to climb, signaling the contamination of the subset with functional reactions (latent positives). The spy-determined threshold (vertical dotted line) effectively identifies the inflection point prior to this contamination, maximizing the inclusion of true negatives while maintaining the structural integrity of the RN subset.

Using the spy-determined threshold, data points below this threshold are relabeled as RN, while any data points predicted by the classifier to have a probability above this threshold are discarded as undecisives. The confusion matrix in Figure 7A shows the performance of our PAYN strategy in identifying the RN. The matrix confirms that the PAYN framework successfully excludes the majority of the latent positives in the unlabeled set. This leads to a high average negative precision of 91% regarding the RN. Overall, 66% of the latent negative data is correctly identified as RN. While the false positive rate of 14.2% is high, lowering it would come at the cost of precision (Figure 6). Comparable conclusions can be drawn from the other data sets (Figure 7B and SI, section 5.4), which provide further evidence to support these findings.

**Figure 7:**
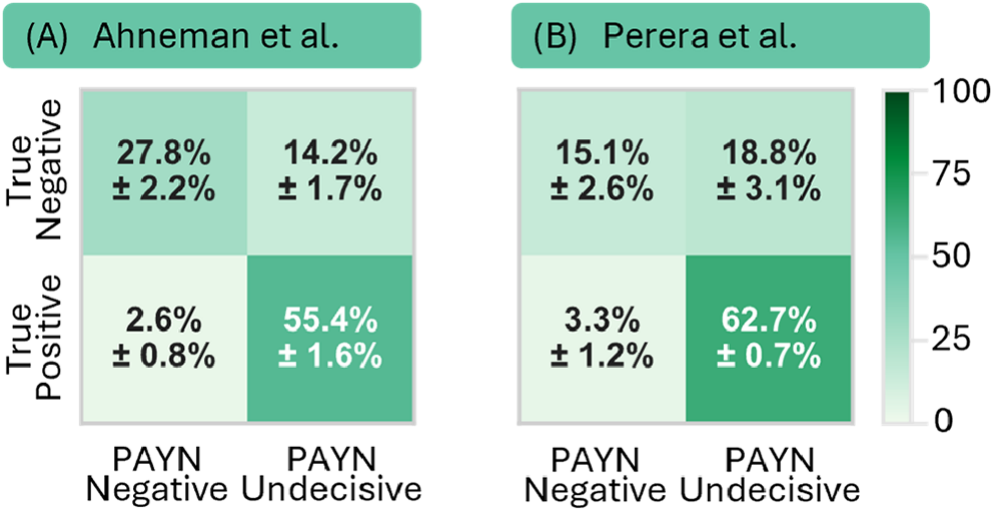
Confusion matrices illustrating the partitioning of the unlabeled data for (A) Ahneman et al. and (B) Perera et al. The matrices contrast the hidden ground-truth labels against the designations assigned by the PAYN classifier. Values indicate the percentage of the total unlabeled population, averaged over five folds (±standard deviation).

### Downstream Application: Yield Prediction

Having rigorously validated the PAYN framework’s capacity to extract high-fidelity RN from the unlabeled distribution, we next assessed the impact of this augmentation on downstream applications. Specifically, we investigated whether a regression model trained on the augmented data set—comprising the original labeled positives and the newly identified RN could effectively mitigate the bias inherent to positive-only training. The central objective was to determine if the inclusion of these inferred negatives could bridge the substantial performance gap that typically separates models trained on sparse, success-biased literature data from those trained on comprehensive, fully labeled HTE campaigns. To contextualize the efficacy of the model trained on PAYN augmented data, we established two distinct benchmark models that we trained in addition to our augmented regression model:

1. Upper Benchmark (Fully labeled): First, a standard regression model was trained and optimized on the fully labeled data set (containing known yields for all examples). This represents an idealized, data-rich scenario—typically achievable only after extensive HTE campaigns—and serves as the performance upper bound. As the augmented model utilizes only a fraction of these ground-truth labels alongside unlabeled data, it is expected to approach, but not exceed, this benchmark.
2. Lower Benchmark (Positives only): Conversely, we trained a “positives-only” model using exclusively the labeled positive entries available to the augmented model, ignoring the unlabeled data entirely. This baseline mimics the prevailing scenario in chemical literature. Models trained on this data often fail to predict negative outcomes on unseen data and remain ineffective for application. Because we hypothesize that by including RN this issue might be resolved, the positive-only model serves as the lower performance bound.

To ensure comparability, all three models—fully labeled, positives only, and PAYN augmented—were trained, optimized, validated, and evaluated on identical data splits. To better understand the generality of our approach, we further included two more combinatorial HTE data sets by Stevens et al. and Perera et al. (Figure 5) and subjected them to comparable analyses, such as discussed so far (SI, section 5). The results are displayed in Figure 8.

**Figure 8:**
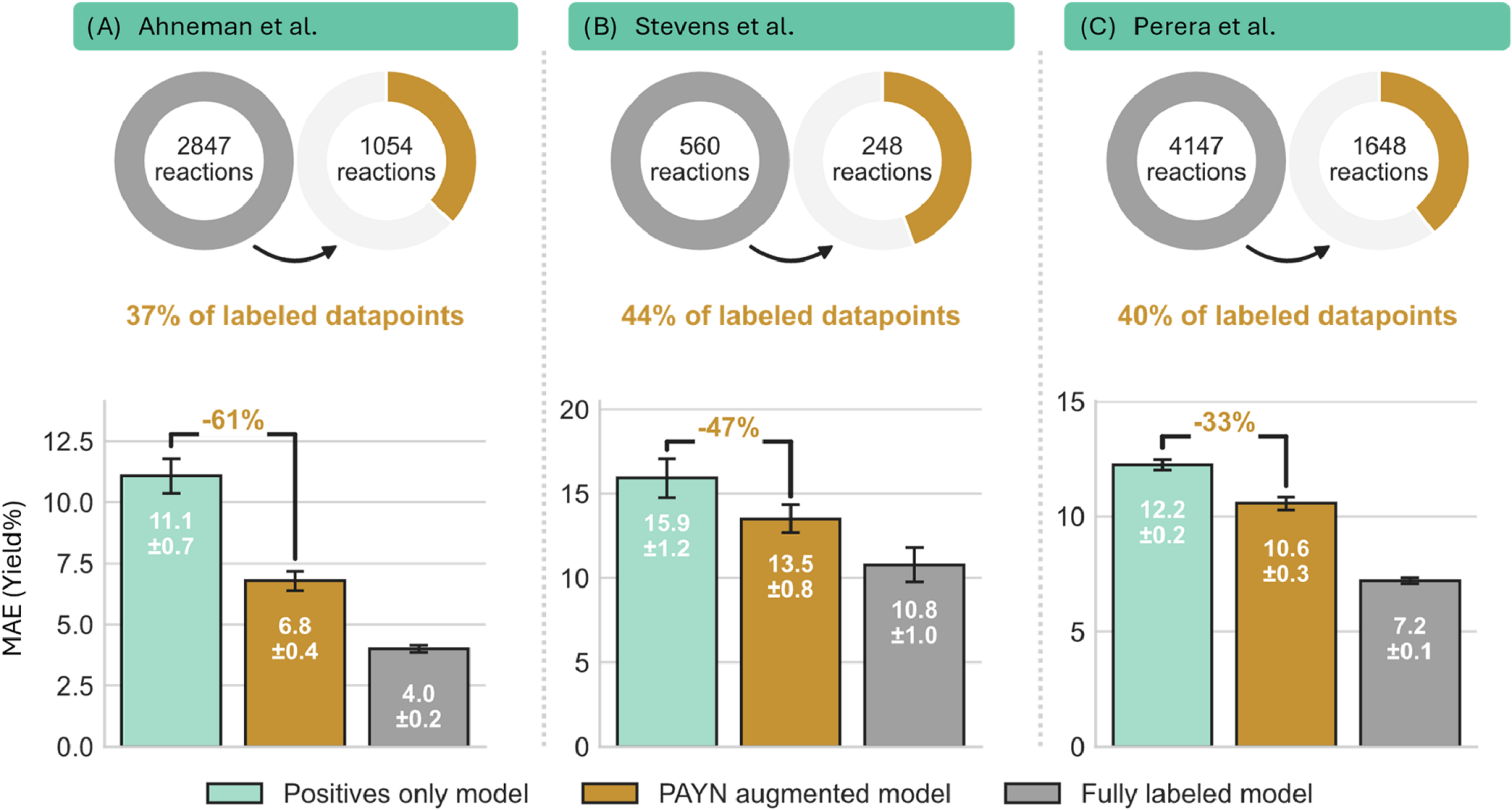
Benchmarking of the PAYN augmented regression model against theoretical performance limits across three HTE data sets. Circles represent the amount of labeled data points used by the PAYN augmented model and positives only model in comparison to the fully labeled model. The bar chart displays the MAE for the positives only lower benchmark (light green), the proposed PAYN augmented model (gold), and the fully labeled upper benchmark (gray). Annotated percentages indicate the proportion of the performance gap between the positives only and fully labeled models that is successfully bridged by the PAYN framework. Error bars represent the standard deviation across five cross-validation folds.

In all cases, the augmented model outperforms the positives-only baseline, confirming our hypothesis that the unlabeled portion of the data set contains latent structural information critical for defining the reaction landscape. The most pronounced improvement was observed in the data set by Ahneman et al., where the positives-only model yielded a baseline MAE of 11.1%. The inclusion of the spy-determined RN via the PAYN framework reduced the MAE to 6.8%. When viewed in the context of the fully labeled upper bound (MAE of 4.0%), our approach successfully bridged 61% of the performance gap, without the need for any additional experiments and utilizing only 37% of the labeled data. The performance difference toward the fully labeled model is partially also attributed to the smaller data set size, which is further discussed in the SI, section 5.8.

Similar trends were observed for the Stevens et al. and Perera et al. data sets, with performance improvements of 47% and 33% (Figure 8B,C), respectively. Since the variance in performance between different folds is high (Figure 8B), we conducted a Wilcoxon signed-rank test that shows that the augmented model statistically outperforms the positive-only model (SI, section 5.9). We attribute the variation in performance gain to the underlying topology of the chemical spaces, as well as the density and size of the HTE data sets. For ease of use and better comparability, all variable components of the combinatorial HTE sets (e.g., substrates, ligands, bases, etc.) were encoded using the ECFP (SI, section 3 for further details). Likely, there is a higher overlap between the successful and failed reactions in the relevant feature space of the Perera et al. data set, than in the Ahneman et al. data set. We thus hypothesize that a more problem-tailored featurization might close more of the performance gap.

Nevertheless, the consistent reduction in error across all benchmarks demonstrates that the PAYN framework is robust. Even in the most challenging case (Perera et al.), the model leveraged unlabeled data to achieve an MAE 1.6% absolute reduction and the ability to predict negative outcomes compared to the literature-standard positives-only approach (SI, section 5.7 for scatter plots), a nontrivial improvement in the context of yield prediction.

Thus far, we have only examined combinatorial HTE data sets that densely cover a small predefined chemical space of a few thousand chemical reactions. The parameters of these data sets were also picked using chemical intuition to find optimal conditions within this space, so a certain selection bias is inherent to them. This is evident by the large proportion of positives that still remain within them (SI, section 3). We thus decided to test our PAYN framework on one more recently published data set by Neves et al., that was designed to cover a huge reaction space of 11.5 M possible reactions.^46^

Contrary to the previous data set, the 11 328 reactions executed to create this data set were selected to maximize diversity across this chemical space. The stark difference to the other data sets is evident in the low proportion of positively classified reactions, accounting for less than 29% of the data set (Figure 9).

**Figure 9:**
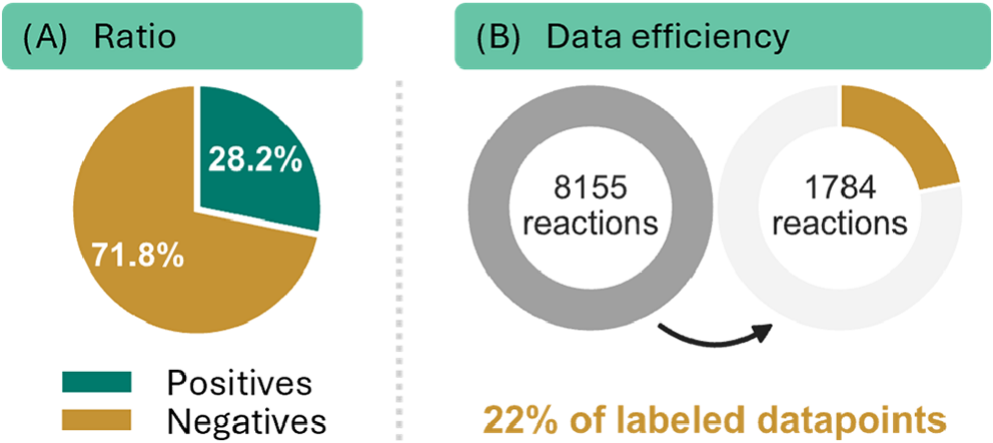
(A) Ratio of positive and negative reactions within the Neves et al. data set based on a yield threshold of 20%. (B) Circles represent the amount of labeled data points used by the PAYN augmented model and positives only model in comparison to the fully labeled model.

By using only positive data points and 22% of the overall data points, we were able to close the performance gap between the positives only (MAE 27.9%) and the fully labeled model (MAE 7.05%) by 75%, achieving an MAE of 12.3%. Despite the unlabeled data set already consisting of mostly negative data points, PAYN is still able to increase the proportion of negatives to up to 97% in the RN.

## Conclusions

In this study, we demonstrate that even severely biased data can be leveraged to train robust ML models for yield prediction in chemistry. We derived these biased data sets from fully labeled HTE data sets by simulating reporting bias that exceeds that found in literature data. Even with the complete removal of all negative data points within these data sets, we show that our PAYN framework successfully applies PU learning to generate reliable negatives. The framework’s generality and scalability were validated across diverse reaction types—including Ni-catalyzed borylations, Buchwald–Hartwig, and Suzuki–Miyaura couplings, and data sets of varying scales.

We hope that our findings support future applications of the vast and valuable resource of literature data for yield predictive models. By utilizing the spy technique, PAYN prioritizes the precision of reliable negatives. This minimizes the risk of misclassifying latent positives as failures, thereby preventing the algorithmic exclusion of potentially successful chemical pathways in downstream applications. The computational generation of reliable negatives enables the construction of balanced data sets. We demonstrated the use of these augmented data sets for the training of yield predictive models, showcasing a performance approaching the accuracy of models trained on fully labeled ground-truth data.

The fundamental principles established here suggest that chemistry, where successful reactions are rare islands in a vast space of nonreactivity, is uniquely amenable to PU learning. We envision the integration of such recovered chemical knowledge into future active learning and Bayesian optimization campaigns, potentially accelerating the “cold start” of such workflows while significantly reducing the consumption of resources and labor. We hope to facilitate the adoption of PU learning strategies in the community with our open-source Python framework while simultaneously advocating for FAIR (Findable, Accessible, Interoperable, Reusable) data principles to ultimately resolve the reporting bias at its source.

## Supplementary Material



## Notes

Code and data availability, analysis of literature and HTE data sets, PAYN framework description and workflow, hyperparameter optimization, and further experimental results (PDF)

## Abbreviations

PAYN: positivity is all you need
HTE: high-throughput experimentation
ML: machine learning
PU: positive-unlabeled
SCAR: selected completely at random
RN: reliable negative
ECFP: extended-connectivity fingerprints
MFF: multiple fingerprint features
DFT: densitiy functional theory
MAE: mean average error.
