# Supplementary material for: Yield Prediction of Organic Reactions in Biased Data Sets via Positive-Unlabeled Learning
Source: J Am Chem Soc. 2026 Apr 1;148(14):15066–75. doi: 10.1021/jacs.6c00127 (PMC13088182; doi:10.1021/jacs.6c00127)
Supplement: Supplementary file 1 [file ja6c00127_si_001.pdf]

Supplementary Information

Yield Prediction of Organic Reactions in Biased Datasets via Positive-Unlabeled Learning

Florian Boser‡, Jan C. Spies‡ and Frank Glorius*

Organisch-Chemisches Institut, Universität Münster, Münster, Germany.

‡These authors contributed equally.

*Correspondence to: glorius@uni-muenster.de

TABLE OF CONTENTS

1. CODE AND DATA AVAILABILITY 3

2. ANALYSIS OF LITERATURE DATA 3
   - 2.1. Reaxys Search and Data Export 3
   - 2.2. Data Cleaning 3
   - 2.3. Visualization of Yield Distribution 3

3. HTE DATSETS FROM LITERATURE 4
   - 3.1. Analysis of Dataset 1 4
   - 3.2. Analysis of Dataset 2 5
   - 3.3. Analysis of Dataset 3 6
   - 3.4. Analysis of Dataset 4 7

4. PAYN FRAMEWORK 8
   - 4.1. Configuration Management 8
     - 4.1.1 Loading Configuration (*payn.ConfigLoader.ConfigLoader)* 8
     - 4.1.2 ArgParser for CLI / Slurm (*payn.ConfigLoader.ConfigArgParser*) 8
   - 4.2. Logging (*payn.Logging.Logger*) 9
   - 4.3. Data Loader (*payn.DataLoader.DataLoader*) 9
   - 4.4. Data Schema & Integrity Verification (*payn.DataSchema.DataSchema*) 9
   - 4.5. Featurization (*payn.Featurisation.Featurisation*) 10
     - 4.5.1 Multi-Feature Fingerprinting (MFF) Reimplementation (*payn.Featurisation.MFFFingerprinter*) 10
   - 4.6. Splitting of Dataset (*payn.Splitting.DataSplitting*) 11
   - 4.7. Spy Injection and PU Generation (*payn.Splitting.SpySplitting*) 11
   - 4.8. Spy Model (*payn.AugmentationModels.SpyModel.SpyModel*) 11
   - 4.9. Yield Prediction Regression Model (*payn.RegModel.RegModel*) 12
   - 4.10. Hyperparameter Optimization (*payn.Optimization.Optimization*) 12
     - 4.10.1 Bayesian Optimization (*payn.Optimization.BayesianOptimization*) 13
     - 4.10.2 Grid Optimization (*payn.Optimization.GridOptimization*) 13
   - 4.11. Reliable Negative Identification (*payn.AugmentationModels.SpyModel.augmen_negative_identifier*) 13
   - 4.12. Data Recombination (*payn.Recombination.DataRecombination*) 14
   - 4.13. Evaluation (*payn.Evaluation.Evaluator*) 14
   - 4.14. Visualisation (*payn.Visualisation.Visualisation*) 15
   - 4.15. Utilities (*payn.Utility*) 15
   - 4.16. Tests (*tests*) 16

5. EXPERIMENTS 16
   - 5.1. General Hyperparameters 16

5.2. Determining the Best Metric for the PU Classifier 17
5.3. Analyzing the Effects of Spy Rate and Spy Tolerance 19
5.4. Investigating Smoothness and Separability 23
5.5. Analysis of the Reliable Negatives 24
5.6. Benchmarking of the Augmented Regression Model 24
5.7. Comparison of Predictions in Regression Models 25
5.8. Impact of Dataset Size on Performance of the Fully Labeled Model 27
5.9. Statistical Analysis of the Augmented Model 30

6. REFERENCES 32

1. CODE AND DATA AVAILABILITY

The framework used within this work is available free of charge at https://github.com/GloriusGroup/PAYN. The datasets used within this work are openly available: Ahnemann et al.[1], Stevens et al.[2], Perera et al.[3] and Neves et al.[4] The datasets and further experimental data is available at zenodo: https://doi.org/10.5281/zenodo.18609343.

2. ANALYSIS OF LITERATURE DATA

2.1. Reaxys Search and Data Export

All data was exported from Reaxys between the 23rd to the 24th of October 2024. Several search queries were used (see Figure S1) to sample all possible results for Suzuki-Miyaura cross couplings within the Reaxys database.[5] The search results were then exported via the native export functionality of Reaxys.

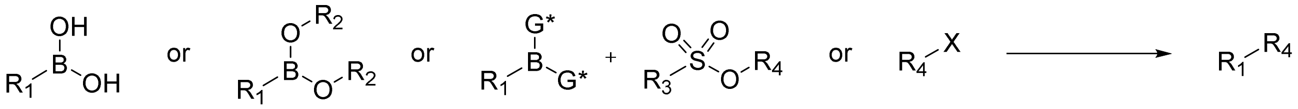

R_1, R_2, R_3 = ALK, AEL, AYL, ARY, HAR

R_4 = AEL, AYL, ARY, HAR

Figure S1: Schematic depiction of queries used to extract Suzuki cross coupling reactions from Reaxys.

2.2. Data Cleaning

All entries which did not have a yield value, reaction SMILES, or reagent were dropped. Furthermore, each reaction SMILES was inspected to include exactly two starting materials and one product SMILES string.

2.3. Visualization of Yield Distribution

The yield distribution of the extracted data is shown in Figure S2. A clear trend towards higher yields is apparent with 40% of all datapoints having a yield of above 80% and only 4% of all datapoints being below 20%. These results are in line with literature datasets previously described in literature.[6,7] This is in stark contrast to the yield distribution found in the HTE datasets used within this work (see Section 3) and has been attributed to reporting and selection bias.

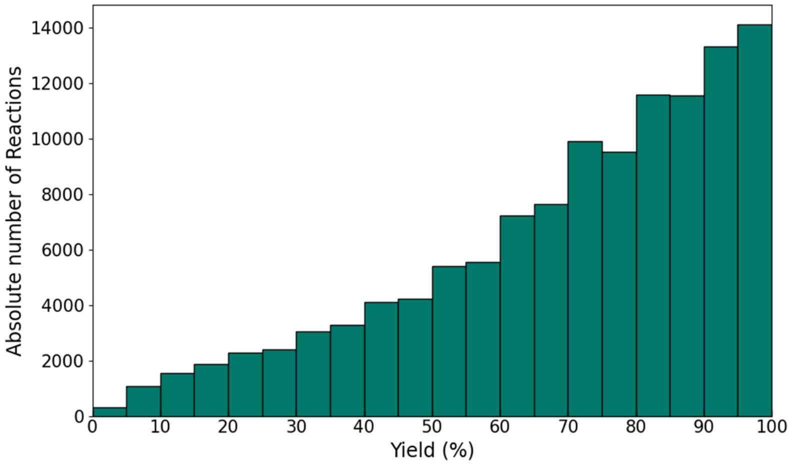

Figure S2: Yield distribution of Suzuki-Miyaura reactions sampled from the Reaxys database.[5]

3. HTE DATSETS FROM LITERATURE

For all datasets (if not stated otherwise), all components of the combinatorial space (Substrates, Ligands, Bases, Solvents, etc.) were provided as SMILES strings. The components that make up the combinatorial space of each dataset are stated in the following sections. From all components the ECFP was generated, concatenated to a single vector and finally bit condensation performed (see section 4.5).

3.1. Analysis of Dataset 1

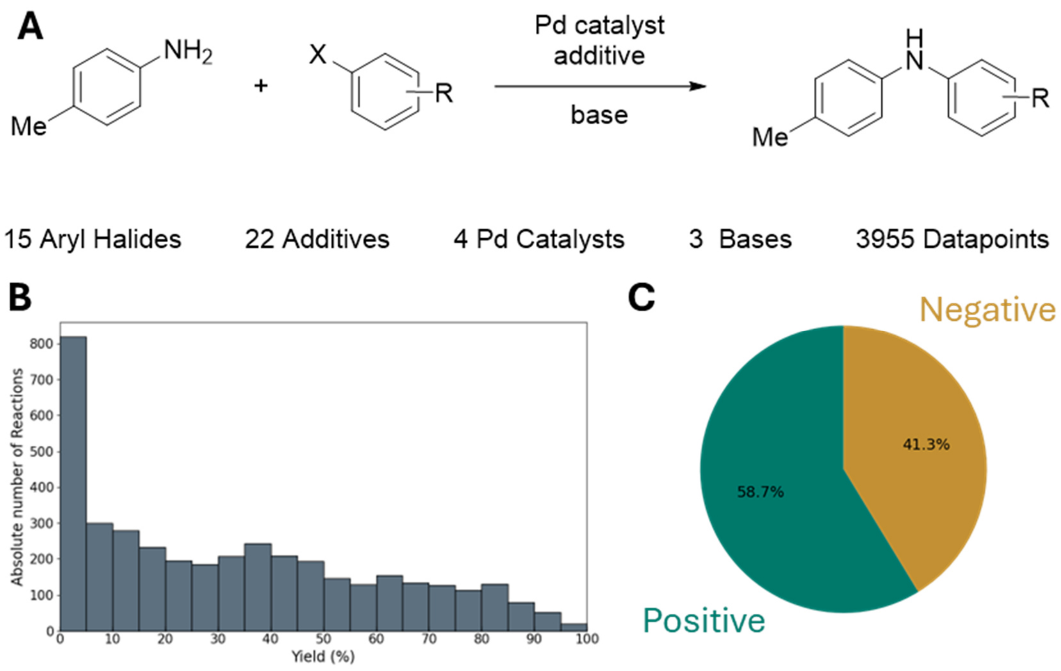

Figure S3: Overview of the Buchwald-Hartwig HTE benchmarking dataset reported by Ahnemann et al.[1] (A) General reaction scheme and parameter space dimensions. (B) Histogram of reaction yields. (C) Class distribution (Positive/Negative) based on a 20% yield threshold.

The dataset published by Ahnemann et al.[1] comprises 3955 distinct Buchwald-Hartwig C–N cross-coupling reactions. After bit condensation (see SI section 4.5) the final feature vector has a length of 389 bits. The combinatorial space includes the coupling of 4-methylaniline with 15 aryl halides, utilizing a diverse set of 22 additives, 4 palladium catalysts, and 3 bases. As shown in the yield distribution (Figure S3B), while reactions resulting in 0-5% yield are the most frequent, the majority of the dataset is high yielding. Using a binary classification threshold of >20% yield, 58.7% of the data points are labeled as positive (Figure S3C).

3.2. Analysis of Dataset 2

The dataset published by Stevens et al.[2] comprises 779 distinct borylation reactions of aryl (pseudo-)halides. After bit condensation (see SI section 4.5) the final feature vector has a length of 433 bits. The combinatorial space includes 33 aryl (pseudo-)halides that were borylated using Ni(II) with one of 23 ligands. It is worth noting that in the original publication, each reaction was executed in both MeOH and EtOH. However, for this work only datapoints with EtOH as solvent were used. As shown in the yield distribution (Figure S4B), while reactions resulting in 0–5% yield are the most frequent, the majority of the dataset is high yielding. Using a binary classification threshold of >20% yield, 76.9% of the data points are labeled as positive (Figure S4C).

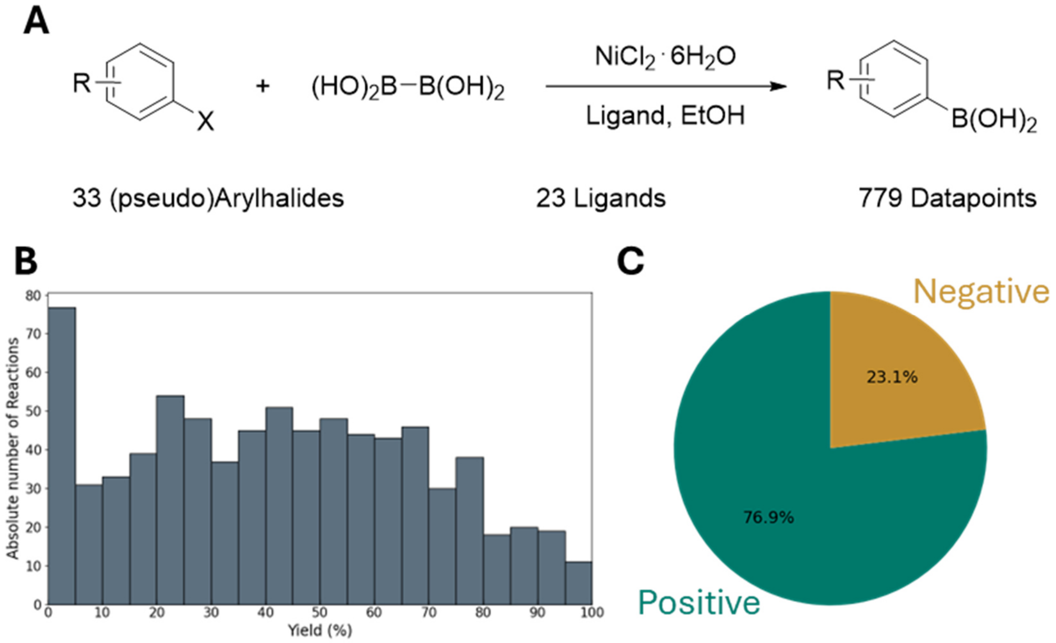

Figure S4: Overview of the Ni-catalyzed borylation HTE dataset reported by Stevens et al.[2] (A) General reaction scheme and parameter space dimensions. (B) Histogram of reaction yields. (C) Class distribution (Positive/Negative) based on a 20% yield threshold.

3.3. Analysis of Dataset 3

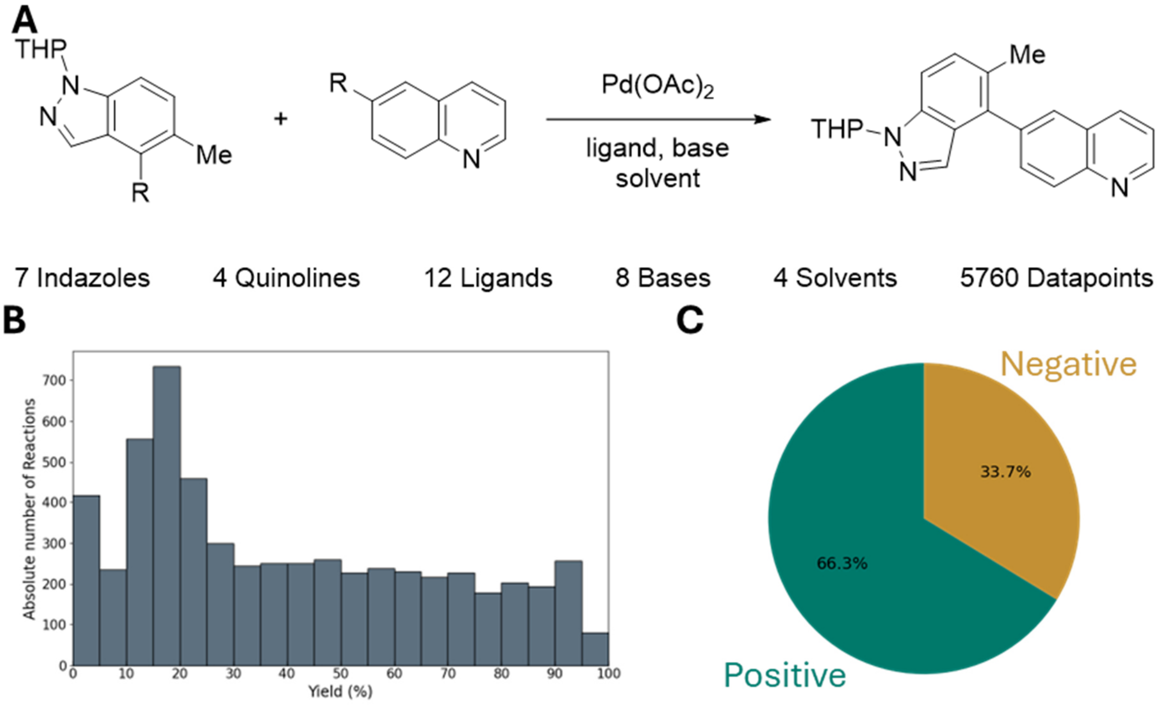

Figure S5: Overview of the Suzuki-Miyaura cross-coupling HTE dataset reported by Perera et al.[3] (A) General reaction scheme and parameter space dimensions. (B) Histogram of reaction yields. (C) Class distribution (Positive/Negative) based on a 20% yield threshold.

The dataset published by Perera et al.[3] comprises 5760 distinct Suzuki-Miyaura cross-coupling reactions. After bit condensation (see SI section 4.5) the final feature vector has a length of 275 bits. The combinatorial space includes the coupling of 7 indazoles and 4 quinolines, using one of 12 ligands, 8 bases and 4 solvents leading to an extensive set of 384 unique reaction conditions. As shown in the yield distribution (Figure S5B), low yielding reactions resulting in 10–25% yield are the most frequent, while the majority of the dataset is high yielding. Using a binary classification threshold of >20% yield, 66.3% of the data points are labeled as positive (Figure S5C).

3.4. Analysis of Dataset 4

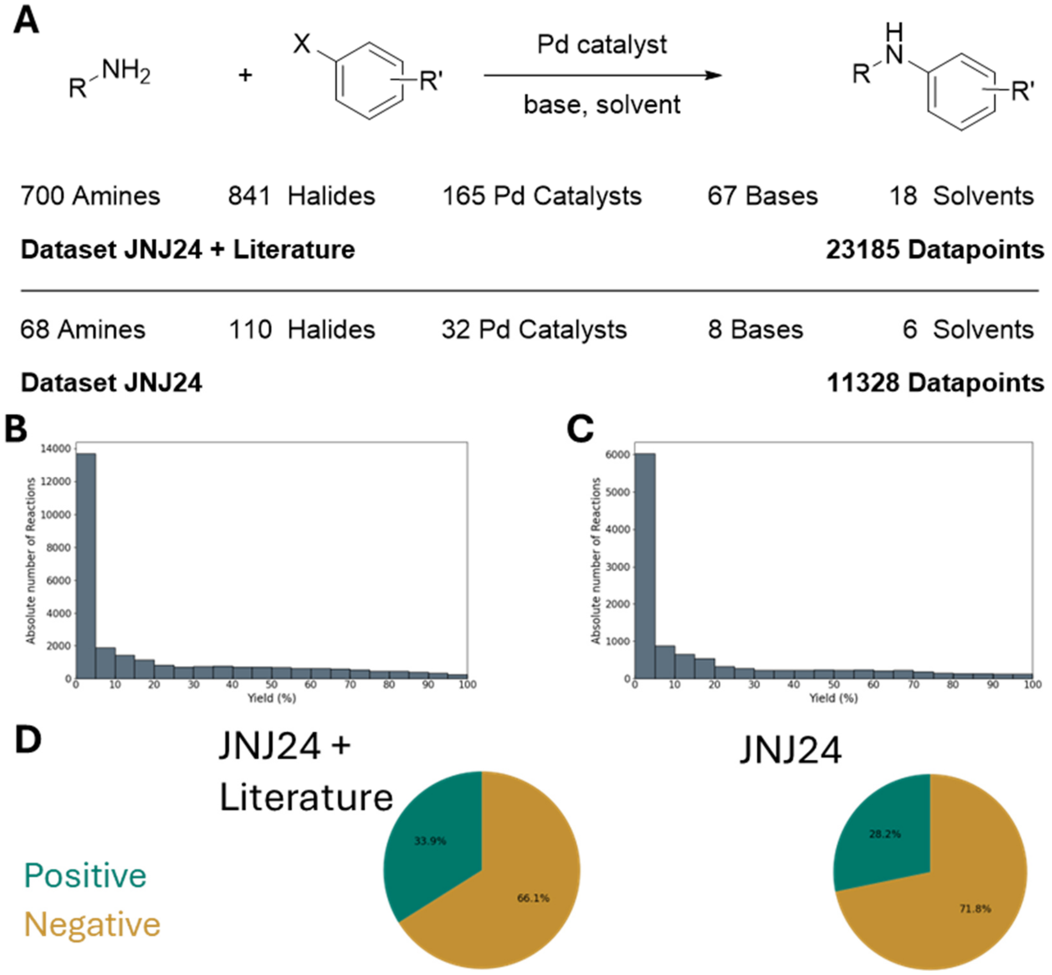

Figure S6: Overview of the Buchwald-Hartwig HTE dataset reported by Neves et al.[4] The 'JNJ24' dataset only comprises new datapoints, while the 'JNJ24 + Literature' dataset also contains previously published datasets. (A) General reaction scheme and parameter space dimensions. (B) Histogram of reaction yields for 'JNJ24'. (C) Histogram of reaction yields for 'JNJ24 + Literature'. (D) Class distribution (Positive/Negative) of both datasets based on a 20% yield threshold.

The dataset published by Neves et al.[4] was constructed by sampling various previous Buchwald-Hartwig datasets and combining them. A data-guided approach was used to select new data points to increase the diversity of the dataset and improve the final model's overall performance. These data points were generated through HTE. The final dataset consists of 23185 datapoints (including the literature datasets, herein referred to as 'JNJ24 + Literature'), while 11328 datapoint were newly generated (herein referred to as 'JNJ24'). While the original 'JNJ24 + literature dataset also contains the Ahnemann et al. data, these datapoints were dropped. Interestingly, the yield distributions of both datasets show a much higher shift towards very low yielding results. Also, for these two datasets the majority of datapoints are below the classification threshold of 20% (Figure S6D). This might be due to selection biases on account of the previously discussed HTE datasets. Typically, the chemical space of a HTE campaign is selected not only to maximize diversity, but also to identify high-yielding conditions. While for all previous datasets the data was featurized using the native PAYN featurization

(see section 4.5), for this dataset the provided DFT features or the DRFP were used, without bit condensation.

4. PAYN FRAMEWORK

The PAYN framework is an open-source Python library for the execution and evaluation of Positive-Unlabeled (PU) learning in organic chemistry (available on GitHub at https://github.com/GloriusGroup/PAYN). PAYN is implemented in Python3, building upon the core scientific stack including pandas, RDKit, CatBoost, Optuna, numpy and sklearn among others.[8–14] The architecture enforces a separation of concerns between configuration, data featurization, model training, and evaluation to ensure reproducibility and extensibility. Below we detail the core modules responsible for experiment orchestration. We also refer readers to the extensive documentation available within the repository (https://GloriusGroup.github.io/PAYN).

4.1. Configuration Management

Centralized configuration management is employed to ensure that experimental conditions are explicitly defined and reproducible. PAYN utilizes a hierarchical configuration file (*YAML* or *JSON*) to ensure all experimental conditions—from data paths to hyperparameter search spaces—are explicitly defined and version-controllable.

4.1.1 Loading Configuration (*payn.ConfigLoader.ConfigLoader)*

The *ConfigLoader* module serves as the primary interface for state initialization.

- **File Parsing:** It handles the ingestion of static configuration files, supporting both *YAML* and *JSON* formats with automatic type inference.
- **Type Safety:** The loader parses the raw input into structured Python dictionaries, acting as the single source for experimental parameters.
- **Instantiation Factory:** Key classes (e.g., *Featurisation*, *Optimisation*) implement a *from_config* class method. This "Config-as-Code" pattern decouples the class logic from the configuration structure, allowing classes to be instantiated programmatically during testing while remaining easily properly configured by the global *config* during production runs.

4.1.2 ArgParser for CLI / Slurm (*payn.ConfigLoader.ConfigArgParser*)

To facilitate high-throughput computing (HTC) and integration with workload managers (e.g., Slurm), the *ConfigArgParser* class enables dynamic runtime modification of the configuration.

- **Dynamic Argument Generation:** The module recursively traverses the loaded configuration dictionary and automatically generates a corresponding command-line interface (CLI) argument for every parameter (e.g., nested keys like *spy_model.eval_metric* are mapped to flags like --*spy_model_eval_metric*)
- **Runtime Overrides:** This allows specific parameters to be modified for individual jobs within a batch array without altering the source configuration file, ensuring that the base experimental structure remains constant while variables (e.g. hyperparameters) can be altered.

- **Provenance:** All CLI overrides are logged explicitly at the start of the run to ensure the exact set of parameters used for a specific job can be reconstructed.

4.2. Logging (*payn.Logging.Logger*)

Serves as the centralized logging of the pipeline, interfacing directly with MLflow and Optuna to ensure full experiment reproducibility and transparency. Abstracts the complexity of artifact serialization (DataFrames to CSV buffers) and hyperparameter tracking.

- **Parameter Logging:** The *log_model_hyperparameters* function differentiates between user-defined overrides and model defaults, logging both (defaults prefixed with *config_*) to clarify exactly which parameters drove the model's behavior.
- **Artifact Serialization:** Utilizes StringIO buffers to log pandas DataFrames directly to MLflow without creating intermediate temporary files on disk, reducing I/O overhead and filesystem clutter.
- **Reproducibility:** Logs the exact state of the Optuna study (trials, best parameters, durations) and the resulting model attributes (feature importances, best iteration), allowing for the reconstruction of the optimization trajectory.
- **Data Snapshots:** Captures specific slices of the data at critical checkpoints (e.g., *log_spysplit_data*, *log_augmen_negatives*) to verify the correctness of the PU split logic post-execution.

4.3. Data Loader (*payn.DataLoader.DataLoader*)

Provides a unified abstraction layer for data ingestion. It decouples the downstream pipeline from specific file formats, automatically inferring the correct parsing strategy (CSV vs. Excel) based on file extensions.

- **Dynamic:** The class accepts dynamic keyword arguments *(**kwargs*), allowing users to pass format-specific parameters (such as sheet_name for Excel or delimiter for CSV) directly from the central configuration file without requiring code changes.

4.4. Data Schema & Integrity Verification (*payn.DataSchema.DataSchema*)

Serves as a template for data structures within the pipeline. It dynamically creates the expected schema (column names for features, metadata, and labels) based on the configuration files provided by the user and decouples the code from specific column names in the dataset.

- **Dynamic Validation:** The *validate_dataframe* function enforces schema compliance at runtime, ensuring that required columns (derived from *config.yaml*) are present before computationally expensive operations begin.
- **Modes:** Distinguishes between training mode (requires ground truth labels) and inference mode

(relaxed constraints).

- **Leakage Prevention:** The *verify_no_leakage* and *validate_split_integrity* utilities enforce strict index-based checks. They mathematically verify that training, validation, and test sets share no overlapping indices (data leakage) and that the union of split indices exactly matches the input dataset (data conservation).

4.5. Featurization (*payn.Featurisation.Featurisation*)

Orchestrates the transformation of chemical identifiers (SMILES) into machine-readable numerical vectors. It supports a hybrid approach, allowing the generation of new fingerprints (ECFP, MFF) alongside the ingestion of pre-calculated molecular descriptors (e.g., DFT properties).

- **Bit Condensation:** Implements a *condensed_bits* function that automatically identifies and removes bit positions with zero variance across the dataset, reducing feature sparsity and dimensionality without information loss.
- **Deterministic Featurization:** The explicit transform pipeline ensures that feature vectors are generated identically for every run given the same configuration.
- **Binary Classification:** The pipeline includes a binary classification wrapper (*classify_binary*) that strictly maps continuous regression targets (e.g., yield %) to binary labels (Negative/Positive) based on a fixed threshold.

4.5.1 Multi-Feature Fingerprinting (MFF) Reimplementation (*payn.Featurisation.MFFFingerprinter*)

Reimplementation of the feature generation strategy from EasyChemML designed to maximize information capture by stacking diverse cheminformatics descriptors.[15] The MFF vector is a concatenation of the following molecular representations, computed using RDKit:

- **Connectivity:** RDKit Fingerprints (branched and linear paths, lengths 2, 4, 6, 8).
- **Circular:** ECFP fingerprints (radii 0, 2, 4, 6), computed both with standard invariants and feature-based invariants.
- **Substructure:** Layered Fingerprints (max paths 2, 4, 6, 8) and MACCS Keys (167 bits).
- **Topology:** Avalon, Atom Pairs, and Topological Torsion descriptors.**:**

The final vector length is dynamic, determined by the base *bit_length* parameter (default 2048) multiplied by the 22 variable-length components, plus the fixed-length MACCS keys. A local caching mechanism (*_fingerprint_cache*) ensures that identical SMILES strings within a dataset yield identical vectors without redundant computation. Missing data (flagged via *absence_flag*) is deterministically mapped to a zero-vector of the exact expected length, preserving matrix shape integrity.

4.6. Splitting of Dataset (*payn.Splitting.DataSplitting*)

Provides reproducible mechanisms for partitioning data into training, validation, and test sets. It prevents data leakage by ensuring total isolation of indices across splits. The following splitting strategies are implemented:

- **Random Split (K-Fold/Single):** Standard stratified shuffling (with random seed from *config*) for general-purpose evaluation. 5-fold splitting is our default within this work.
- **Scaffold Split (Leave-One-Group-Out):** Partitions data based on molecular scaffolds (core structures). This evaluates the model's ability to generalize to structurally distinct families of molecules.
- **Butina Clustering Split:** Uses RDKit's Butina algorithm to cluster molecules based on Tanimoto similarity (using fingerprints). Entire clusters are assigned to either train or test sets to ensure that the test set is chemically distinct from the training set, enforcing a rigorous test of out-of-domain generalization.

All splitting methods are tightly integrated with *payn.DataSchema* validators to automatically verify split integrity (conservation of row counts, mutual exclusivity of indices) before any training occurs.

4.7. Spy Injection and PU Generation (*payn.Splitting.SpySplitting*)

Transforms the standard fully labeled dataset (Positive/Negative) into a Positive-Unlabeled (PU) format suitable for the spy technique. It simulates a scenario where only a subset of positives are known, and the rest are hidden within a pool of unlabeled data. Two different PU partitioning strategies are implemented:

- **Controlled Ratio (*split_data_with_controlled_PU_ratio*):** Enforces a strict ratio between known positives and the unlabeled pool by partitioning the dataset and discarding excess negatives. This allows for controlled experimentation on the impact of class imbalance. This partitioning is the default within this work.
- **Original Ratio (*split_data_with_original_PU_ratio*):** Preserves all negative data and mixes in a calculated subset of positives to achieve a target "unlabeled positive concentration".
- **Spy Infiltration:** A user-defined fraction (*spy_rate*, typically 15-20%) of the known positive training set is randomly sampled (deterministically via *random_state*) and moved into the unlabeled set.
  These "spies" have their labels changed to 0 (negative, s = 0) within the model training context but retain their metadata role as unlabeled spy (y = 1). They serve as anchors: since the model *should* have classified them as positive, their predicted probability distribution helps identify other hidden positives and therefore the calculation of a threshold.

4.8. Spy Model (*payn.AugmentationModels.SpyModel.SpyModel*)

Wraps a CatBoost classifier. Selected for its native handling of categorical features and robust performance on tabular chemical data without extensive preprocessing. Other model architectures are applicable here as well, but a class probability score must be calculable or estimable. The spy model is trained on the

spy_infused_training_data (from *payn.SpySplitting*) to distinguish between known positives (s = 1) and unlabeled/spy mixture" (s = 0).

- **Categorical Handling:** The model automatically detects categorical features (e.g., specific bit positions or metadata tags) appended to the end of the feature vector, optimizing the split strategy for mixed data types.
- **Parallelisation:** Automatically detects SLURM cluster environments (SLURM_CPUS_PER_TASK) to adjust thread counts (*thread_count*), ensuring optimal resource usage.
- **Determinism:** Random seeds are propagated strictly from the global *config* to the CatBoost model (*random_state*).
- **Logging:** *SpyModel* (PAYN augmented) is tightly coupled with the *payn.Logging* system. It automatically logs hyperparameters, trained model artifacts, and evaluation metrics (on test sets) to MLflow run immediately after training.

4.9. Yield Prediction Regression Model (*payn.RegModel.RegModel*)

Wraps a CatBoost regressor. Selected for its native handling of categorical features and robust performance on tabular chemical data without extensive preprocessing. Other model architectures are applicable here as well.

- **Parallelisation:** Automatically detects SLURM cluster environments (SLURM_CPUS_PER_TASK) to adjust thread counts (*thread_count*), ensuring optimal resource usage.
- **Determinism:** Random seeds are propagated strictly from the global *config* to the CatBoost engine (*random_state*).
- **Logging:** *RegModel* is tightly coupled with the *payn.Logging* system. It automatically logs hyperparameters, trained model artifacts, and evaluation metrics (on test sets) to MLflow run immediately after training.

4.10. Hyperparameter Optimization (*payn.Optimization.Optimization*)

The *payn.Optimization* module provides a unified interface for hyperparameter tuning, supporting both *CatBoostClassifier* and *CatBoostRegressor* architectures. It ensures consistent model evaluation by wrapping the training logic and enforcing reproducibility constraints across different optimization strategies.

- **Search Space:** The search space is dynamically constructed based on the user configuration (*config.yaml*). The module supports a wide range of CatBoost parameters, including *learning_rate* (log-uniform distribution), *depth* (integer), *iterations* (integer), and multiple additional structural parameters (*grow_policy*, *subsample*, *colsample_bylevel*, *min_data_in_leaf*, *grow_policy*, *one_hot_max_size*, *max_bin*, and *l2_leaf_reg*). By default, in this work *depth* and *learning_rate* were optimized with an early

stopping within 1000 *iterations*.

- **Metric-Aware Directionality:** The module automatically maps the chosen evaluation metric (e.g., *RMSE*, *Logloss*, *F1*, *MCC*) to the appropriate optimization direction (minimize or maximize) using an internal *eval_direction_dict*. This ensures that the objective function correctly rewards or penalizes trial outcomes without manual intervention.

4.10.1 Bayesian Optimization (*payn.Optimization.BayesianOptimization*)

This strategy integrates the Optuna framework to perform efficient exploration of high-dimensional hyperparameter spaces.

- **Tree-structured Parzen Estimator (TPE):** The optimization utilizes a TPE sampler, which models the probability of hyperparameter values given past results. This allows for significantly more efficient traversal of the search space compared to random or grid search methods. In this study, Bayesian Optimization was configured with 50 iterations by default, demonstrating convergence for both spy model and regression tasks.

- **Reproducibility:** The TPESampler is explicitly instantiated with a fixed global *random_state*. This guarantees that the sequence of hyperparameter suggestions is deterministic across experimental runs, eliminating variability caused by the stochastic nature of the sampling process.

4.10.2 Grid Optimization (*payn.Optimization.GridOptimization*)

This strategy implements a combinatorial search over a manually defined grid of hyperparameter values.

- **Combinatorial Search Space:** The module generates every possible combination of the provided parameter lists using Cartesian products (*itertools.product*). This ensures that the global optimum within the defined discrete space is found, provided the grid resolution is sufficient.
- **Deterministic Execution:** Unlike stochastic search methods, Grid Optimization is inherently deterministic.
- **Use Case:** While computationally more expensive than Bayesian methods for high-dimensional spaces, this strategy serves as a robust baseline for validating the stability of specific hyperparameters (e.g., *depth* or *learning_rate*) in isolation.

4.11. Reliable Negative Identification (*payn.AugmentationModels.SpyModel.augmen_negative_identifier*)

This module is the decision-making engine of the PU learning workflow. It leverages the trained Spy Model to filter the unlabeled dataset, identifying a subset of reliable negatives that are statistically distinct from the positive class.

- **Dynamic Thresholding:** Instead of using a fixed probability threshold (e.g., 0.5), the module calculates a dynamic cutoff based on the probability distribution of the spies within the unlabeled datapoints (known positives injected into the unlabeled set). A user-defined *spy_tolerance* (default 5%) % sets the threshold such that 95% of the spies are correctly recognized as positive by the model. This ensures

that the identified negatives are unlikely to be latent positives. Unlabeled data points scoring below this threshold are classified as reliable negatives.

- **Classification**: The module segments the unlabeled data into three distinct categories: 1. Known positives: Original true positives and recovered spies. 2. Reliable negatives: unlabeled data points with predicted probabilities below the calculated threshold. These form the clean negative set for downstream applications such as regression model training. 3. Undecisives: Unlabeled data points with probabilities above the threshold but not labeled as positive. These are discarded to prevent "noisy negatives".

4.12. Data Recombination (*payn.Recombination.DataRecombination*)

This class constructs the final balanced datasets required for downstream tasks, for example yield prediction regression. It merges the verified positive examples with the reliable negative examples identified in the previous step, ensuring the correct assignment of target labels.

- **Yield Assignment:** Known positives retain their experimentally measured yield values (e.g., 20% to 100%) from the original HTE dataset. Augmented negatives are assigned a synthetic yield value (defaulting to 0.0%) to represent reaction failure.
- **Baseline Comparison:** To validate the efficacy of the spy-based filtering, the module also generates a naïve baseline dataset via *generate_unlabeled_as_negative_set*. In this baseline, *all* unlabeled data points are treated as negatives (assigned 0% yield), regardless of their actual likelihood of being latent positives. Comparing the regression performance on the "Augmented" vs. "Positives only" datasets directly quantifies the value added by the PU learning step. Since training on known positives outperformed this approach, we selected the “positives-only” as a comparison for our spy PU approach.
- **Integrity Validation:** Before returning the recombined dataset, the module triggers *validate_split_integrity*. This ensures that the concatenation process has not inadvertently dropped rows or introduced duplicates, preserving the total sample count across the workflow.

4.13. Evaluation (*payn.Evaluation.Evaluator*)

This class provides specialized metrics to assess the quality of the reliable negative identification process. This is possible, due to the use of fully labelled HTE datasets as a ground truth. Since the primary goal of the framework is to construct a balanced training set, standard classification accuracy is insufficient. This module implements negative specific metrics and confusion matrices.

- **Negative-Specific Metrics:** Isolates the subset of data that the model has labeled as reliable negatives and computes negative precision and negative recall. Negative precision measures the purity of the negative set (i.e., *How many of the identified negatives are actually negative?*). High negative precision is crucial to prevent introduction of noise through latent positives. Negative recall measures the coverage (i.e., *What fraction of all true negatives did we successfully find?*).

- **Undecisive Analysis:** The module tracks the volume of undecisive data points—those discarded because their probabilities fell into the zone between the reliable negatives and the known positives.
- **Safety Checks:** The evaluator automatically flags discrepancies, such as "missed negatives" (true negatives that were lost during processing) or index mismatches between the input and output data frames.

4.14. Visualisation (*payn.Visualisation.Visualisation*)

The *payn.Visualisation* module provides a comprehensive suite of plotting tools designed to inspect data distributions and interpret hyperparameter optimization results. Also includes a robust wrapper around Optuna's matplotlib backend to generate a complete portfolio of optimization plots automatically.

- **Yield Distribution Analysis:** The *plot_yield_bins* function generates dual-layer histograms to visualize the spread of target variables (yield). It overlays a standard frequency distribution with a color-coded classification split (positive vs. negative) based on a user-defined threshold. This allows for rapid visual assessment of dataset imbalance and class separability.
- **Automated Artifact Generation:** Upon completion of an optimization study, the system auto-generates key diagnostic plots, including *Optimization History* (convergence tracking), *Hyperparameter Importance*, and *Parallel Coordinate* plots.
- **Pairwise Interaction Analysis:** To reveal complex dependencies between hyperparameters, the module automatically iterates through all parameter pairs to generate *Contour* and *Slice* plots.
- **MLflow Integration:** When enabled, all generated plots are automatically logged as artifacts to the associated MLflow run, creating a permanent visual record of the experiment's search phase.

4.15. Utilities (*payn.Utility*)

This module provides low-level helper functions and classes that support the main execution pipeline.

- **Output Suppression (*utils.py*):** To maintain clean logs in High-Performance Computing (HPC) environments, the package includes *suppress_stdout* and *suppress_output* context managers. These are wrapped around verbose external library calls (e.g., during internal CatBoost initialization) to prevent console spam from burying important application logs.
- **Fine-Grained Experiment Tracking (*callbacks.py*):** The *MLflowCatBoostCallback* class provides an interface for iteration-level logging. Although the main pipeline defaults to logging only final model metrics to conserve storage, this callback can be injected into the training loop to stream metrics (like validation loss per tree) directly to MLflow in real-time.

4.16. Tests (*tests*)

The PAYN framework includes a comprehensive test suite comprising over 125 unit and integration tests, designed to ensure reproducibility. The testing strategy prioritizes determinism, verifying that all stochastic processes (e.g., data splitting, featurization) are bit-for-bit reproducible. Logic isolation is achieved by mocking heavy external dependencies (CatBoost, Optuna, RDKit), allowing for focused verification of orchestration logic such as hyperparameter optimization stability and SLURM threading detection. Additionally, the suite enforces critical data invariants, mathematically validating set disjointness to prevent data leakage and ensuring correct ratio enforcement during PU transformations and custom metric evaluation.

5. EXPERIMENTS

5.1. General Hyperparameters

There is a multitude of hyperparameters that define the PAYN framework, as well as the underlying Catboost models and Optuna optimizer. Most of these were left unchanged over all runs (except if stated otherwise). The general hyperparameter settings that were used can be seen in Table S1.

Table S1: Hyperparameter settings used generally for all experiments in this work.

Hyperparameter	Setting
Random seed	42
Featurisation method	ECFP
ECFP bit length	2048
ECFP radius	2
Condense bits	True
Cross-validation folds	5
Validation set size	0.1
Ratio Positives/Unlabeled	1.0
Regression model evaluation metric	MAE
Optimisation type	Bayesian
Optimization iterations	50
Search space	Depth, learning rate
Maximum depth	12
Minimum learning rate	0.00001
Catboost iterations	1000

5.2. Determining the Best Metric for the PU Classifier

At the first step of the PAYN framework, a Catboost classifier is trained to distinguish between the positive and the unlabeled space. Given the latent positives in the unlabeled set, there is a significant overlap between the feature space of both distributions, making complete separation impossible. Further, while the model is trained to separate the positive and unlabeled distribution, the actual goal of this step is to achieve a separation of the latent positives and negatives within the unlabeled set. The success of this operation can therefore not be quantified by classic classification metrics, making the picking of the optimal one a non-trivial task. We therefore decided to use the performance of the subsequent augmented regression model (MAE) as a read-out, as it is directly influenced by the classifiers ability to extract a large and precise set of Reliable Negatives (RN).

Table S2: Average MAE and ranks of the final augmented regression model over five folds of the PAYN framework, using the Ahneman et al. dataset and various target metrics to train the initial classifier. The average rank was calculated by ranking the results of each fold for each different metric and taking the average over all five folds.

Metric	MAE (Yield %)	STD (Yield %)	Average Rank
PRAUC	6.65	0.46	3.2
AUC	6.64	0.33	3
MCC	6.59	0.31	2.8
F1	7.08	0.53	5.4
Precision	7.64	0.64	7
Recall	6.83	0.37	4
Accuracy	6.56	0.35	2.4

Table S3: Average MAE and ranks of the final augmented regression model over five folds of the PAYN framework, using the Stevens et al. dataset and various target metrics to train the initial classifier. The average rank was calculated by ranking the results of each fold for each different metric and taking the average over all five folds.

Metric	MAE (Yield %)	STD (Yield %)	Average Rank
PRAUC	14.3	1.1	3.6
AUC	13.2	0.8	2.2
MCC	13.5	0.7	2.4
F1	15.3	1.3	5.4
Precision	15.3	1.7	5.4
Recall	14.7	1.1	4.0
Accuracy	15.2	1.5	4.6

Table S4: Average MAE and ranks of the final augmented regression model over five folds of the PAYN framework, using the Perera et al. dataset and various target metrics to train the initial classifier. The average rank was calculated by ranking the results of each fold for each different metric and taking the average over all five folds.

Metric	MAE (Yield %)	STD (Yield %)	Average Rank
PRAUC	10.7	0.32	3.6
AUC	10.8	0.38	4.4
MCC	10.6	0.24	1.8
F1	10.7	0.27	3.2
Precision	11.1	0.25	5.6
Recall	11.0	0.32	5.2
Accuracy	10.8	0.26	4.0

The results of this optimization for all three datasets are shown in Tables S2-S4. Since the actual outcomes of each fold vary widely, the standard deviation is often much larger than the actual differences in the average MAE. We therefore also ranked the performance of each metric for each fold. This also allows for a better comparison between the different models which are displayed in Figure S7. The average rank is displayed for the Ahneman et al. dataset in green, the Stevens et al. dataset in red and the Perera et al. dataset in gold. While there are slight differences in between the different datasets, some general trends are visible. For example, using precision as a target metric for the initial classifier leads to the worst results of the final augmented model over all three datasets. This matches expectations, as the precision metric penalizes the classifier for classifying the latent positives in the unlabeled set as actual positives, effectively forcing the model to treat the entire unlabeled set as definitive negatives. This behavior suppresses the identification of latent positives, thereby contaminating the reliable negative subset. In contrast, the MCC performs best, providing a more balanced assessment, allowing the classifier to better capture the underlying structure of the chemical space rather than overfitting the initial label assignment.

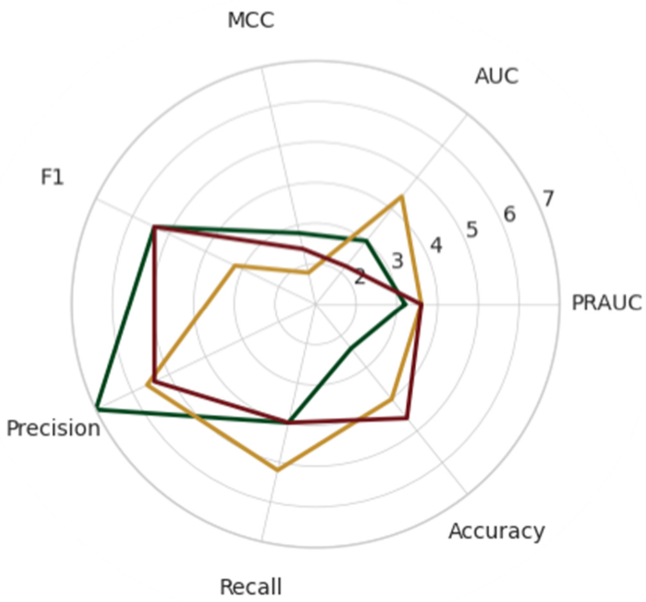

Figure S7: Radar chart of final average ranked performance of the final augmented model when using different classification metrics to optimize the PU Model. Ranked performances are shown for the Ahneman et al. (green), Stevens et al. (red) and Perera et al. (gold) datasets.

5.3. Analyzing the Effects of Spy Rate and Spy Tolerance

The performance of the classifier and the PAYN framework's ability to pick an accurate set of RN is further influenced by the spy rate and spy tolerance. The spy rate is the proportion of all labeled positives that are taken from the population and seeded into the unlabeled set. This proportion needs to be high enough so that the spies taken from the labeled positives properly model the distribution of the whole positive population. At the same time, the larger the number of spies that are taken from the labeled population, the smaller the labeled population gets, and the larger the unlabeled, making it potentially harder for the classifier to learn the feature space of positives.

After training the classifier a threshold is set to determine which part of the unlabeled set is classified as RN. This threshold is determined using the previously added spies. It is assumed that ideally their distribution matches the distribution of the latent positives in the unlabeled set. All datapoints of the unlabeled set are now sorted by their probabilities, as predicted by the classifier, of belonging to the positive class. A threshold is now chosen in a way that only a small portion of the spies would be underneath it. This proportion is called spy tolerance and is necessary to account for potential outliers within the spies and to allow for a trade-off between precision and recall of the RN. If the threshold is set too low, a few spies with low probabilities could shift the threshold, making the number of RN very small. A too high tolerance would lead to many latent positives being falsely classified as RN.

To properly analyze these two hyperparameters and their interaction with one another we performed a thorough grid search. The results of this can be found for all three datasets in Table S5-S7 and are further displayed as heatmaps in Figure S8.

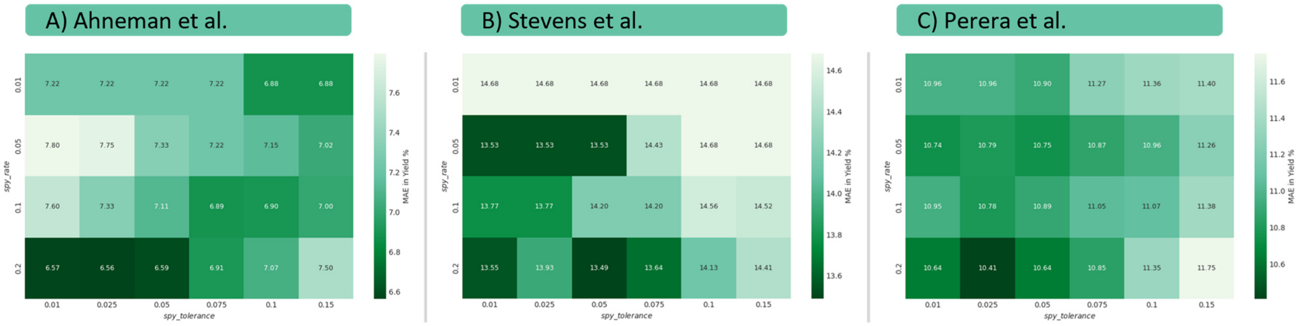

Figure S8: The heatmaps show the average MAE of the final augmented regression model over five folds as a function of *spy_rate* and *spy_tolerance*. The optimization was performed on three distinct HTE datasets (Ahnemann et al., Stevens et al., and Perera et al.). Darker green cells indicate lower MAE and thus superior model performance.

Interestingly, despite the vastly different sizes of the three datasets analyzed, all perform best when a relatively high spy rate of 0.2 is chosen. This might indicate the need for a sufficient subsample of spies needed to accurately model the positive population, which makes up for the need to remove a significant sample of the labeled data. On the other hand, performance decreases with high spy tolerances, which is likely due to a low level of fidelity of the RN. The best performances were reached with spy thresholds around 0.025-0.05, while going lower led to a decrease in performance.

Table S5: Average MAE and ranks of the final augmented regression model over five folds of the PAYN framework, using the Ahneman et al. dataset. A grid search over 24 combinations of spy rate and spy tolerances was performed. The average rank was calculated by ranking the results of each fold for each different metric and taking the average over all five folds.

Spy rate	Spy tolerance	MAE (Yield %)	STD (Yield %)	Average Rank
0.01	0.010	7.22	0.39	13.0
0.01	0.025	7.22	0.39	13.0
0.01	0.050	7.22	0.39	13.0
0.01	0.075	7.22	0.39	13.0
0.01	0.100	6.88	0.31	9.00
0.01	0.150	6.88	0.31	9.00
0.05	0.010	7.80	0.46	22.6
0.05	0.025	7.75	0.36	21.2
0.05	0.050	7.33	0.61	15.4
0.05	0.075	7.22	0.68	11.8
0.05	0.100	7.15	0.46	12.4

0.05	0.150	7.02	0.32	11.2
0.10	0.010	7.60	0.73	20.2
0.10	0.025	7.33	0.72	16.2
0.10	0.050	7.11	0.48	12.6
0.10	0.075	6.89	0.31	9.40
0.10	0.100	6.90	0.33	8.20
0.10	0.150	7.00	0.27	11.8
0.20	0.010	6.57	0.21	3.40
0.20	0.025	6.56	0.28	2.20
0.20	0.050	6.59	0.31	2.60
0.20	0.075	6.91	0.32	9.80
0.20	0.100	7.07	0.30	12.4
0.20	0.150	7.50	0.37	19.0

Table S6: Average MAE and ranks of the final augmented regression model over five folds of the PAYN framework, using the Stevens et al. dataset. A grid search over 24 combinations of spy rate and spy tolerances was performed. The average rank was calculated by ranking the results of each fold for each different metric and taking the average over all five folds.

Spy rate	Spy tolerance	MAE (Yield %)	STD (Yield %)	Average Rank
0.01	0.010	14.7	1.5	12.6
0.01	0.025	14.7	1.5	12.6
0.01	0.050	14.7	1.5	12.6
0.01	0.075	14.7	1.5	12.6
0.01	0.100	14.7	1.5	12.6
0.01	0.150	14.7	1.5	12.6
0.05	0.010	13.5	1.3	8.40
0.05	0.025	13.5	1.3	8.40
0.05	0.050	13.5	1.3	8.40
0.05	0.075	14.4	1.7	12.4
0.05	0.100	14.7	0.73	16.6

0.05	0.150	14.7	0.73	16.6
0.10	0.010	13.8	0.85	9.00
0.10	0.025	13.8	0.85	9.00
0.10	0.050	14.2	1.6	11.0
0.10	0.075	14.2	1.6	11.0
0.10	0.100	14.6	1.3	15.0
0.10	0.150	14.5	0.62	15.2
0.20	0.010	13.5	1.2	8.40
0.20	0.025	13.9	1.6	10.2
0.20	0.050	13.5	0.70	7.80
0.20	0.075	13.6	0.66	8.80
0.20	0.100	14.1	1.0	10.8
0.20	0.150	14.4	0.90	14.6

Table S7: Average MAE and ranks of the final augmented regression model over five folds of the PAYN framework, using the Perera et al. dataset. A grid search over 24 combinations of spy rate and spy tolerances was performed. The average rank was calculated by ranking the results of each fold for each different metric and taking the average over all five folds.

Spy rate	Spy tolerance	MAE (Yield %)	STD (Yield %)	Average Rank
0.01	0.010	11.0	0.63	10.8
0.01	0.025	11.0	0.63	10.8
0.01	0.050	10.9	0.65	8.80
0.01	0.075	11.3	0.57	17.2
0.01	0.100	11.4	0.83	15.6
0.01	0.150	11.4	0.81	16.8
0.05	0.010	10.7	0.14	9.20
0.05	0.025	10.8	0.27	8.80
0.05	0.050	10.7	0.29	6.80
0.05	0.075	10.9	0.32	10.0
0.05	0.100	11.0	0.36	13.0

0.05	0.150	11.3	0.40	18.4
0.10	0.010	10.9	0.35	11.4
0.10	0.025	10.8	0.08	11.0
0.10	0.050	10.9	0.34	10.4
0.10	0.075	11.0	0.36	15.0
0.10	0.100	11.1	0.29	16.0
0.10	0.150	11.4	0.34	20.2
0.20	0.010	10.6	0.36	6.40
0.20	0.025	10.4	0.24	1.40
0.20	0.050	10.6	0.27	6.60
0.20	0.075	10.8	0.30	10.4
0.20	0.100	11.3	0.40	19.6
0.20	0.150	11.8	0.28	23.0

5.4. Investigating Smoothness and Separability

Requirements for PU Learning include smoothness and separability of the data. Smoothness refers to the fact that datapoints which are similar in the feature space should also be similar in the target space. Separability on the other hand requires datapoints of different classes to be distinguishable in the feature space.

We investigated this by calculating the average Tanimoto-Similarity of each datapoint in the Spies, Latent Positives and Unlabeled Negatives to its nearest neighbor in the Known Positive set (see Table S8). While due to the constrained nature of HTE-data the average similarity of all datapoints is quite high, the Spies and Latent Positives show a higher average similarity to the known Positives, than the Unlabeled Negatives. Further, the Spies and Latent Positives show nearly identical similarities to the known Positives. This indicates that both smoothness and separability are given.

Table S8: Average Tanimoto-Similarity to the nearest neighbor in the Known Positive set. The results are averaged over five folds.

Dataset	Spies (Avg. Similarity)	Latent Positives (Avg. Similarity)	Unlabeled Negatives (Avg. Similarity)	Δ (Pos - Neg)
Ahneman et al.	0.85	0.85	0.81	0.04
Stevens et al.	0.68	0.68	0.60	0.07
Perera et al.	0.87	0.86	0.78	0.08

5.5. Analysis of the Reliable Negatives

The fully assigned training dataset for each fold and each literature dataset (Ahneman et al., Stevens et al. and Perera et al.) can be found on zenodo. Each datapoint has its different roles assigned which are: unlabeled positive, unlabeled negative, unlabeled spy, labeled positive, reliable negative and undecisive. Further, using the labels assigned to each unlabeled datapoint and the ground truth label, the ability of the classifier to pick a large and accurate set of RN can be analyzed. In Figure S9 confusion matrixes of each literature dataset are shown which show the average percentage of all unlabeled datapoints, that are correctly or falsely classified as negatives or positives.

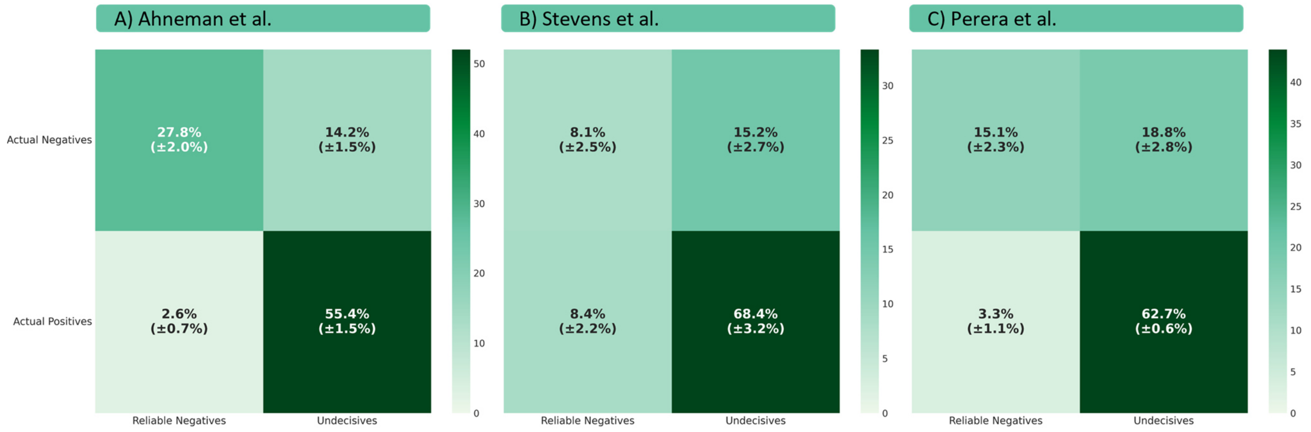

Figure S9: Confusion matrix illustrating the partitioning of the *unlabeled* data for the Ahneman et al., Stevens et al. and Perera et al. dataset. The matrix contrasts the hidden ground-truth labels against the designations assigned by the PAYN classifier. Values indicate the percentage of the total *unlabeled* population, averaged over five folds (± standard deviation).

For all three datasets most of the latent positives were correctly identified as undecisives. In the cases of the Ahneman et al. and Perera et al. dataset there is also a high negative precision leading to RN with a high fidelity. However, this is not the case for the Stevens et al. dataset for which the average RN consists of about 50% of false negatives. In this feature space the catboost classifier cannot separate the latent negatives and positives enough to allow for the picking of high-fidelity RN. Still, the proportion of negatives in the RN is higher than in the unlabeled set and the augmentation of the regression model with these RN leads to a performance boost, despite the low fidelity.

5.6. Benchmarking of the Augmented Regression Model

Using optimized hyperparameters, the classification and subsequent regression models were trained. In addition to these models, also fully labeled and positives only models were trained. In addition to the three previously discussed datasets, also a dataset by Neves et al. was analyzed, using the same hyperparameters. Instead of using the ECFP, the differential reaction fingerprint was used, which is included in the dataset. The average results for each dataset and model can be found in Table S9.

Table S9: Results of the final models trained with optimized hyperparameters. For all datasets MCC was used as a target metric to train the classification model. For the datasets by Ahneman et al., Stevens et al. and Perera et al. a spy rate of 0.2 and spy tolerance of 0.05 were used. For the dataset by Neves et al. a spy rate of 0.01 and a spy tolerance of 0.05 were used. For the Neves et al. dataset only the new datapoints were used (JNJ24).

Dataset	Model	MAE (Yield %)	STD (Yield %)	Improvement
Ahneman et al.	Augmented	6.78	0.39	**61%**
	Fully labeled	4.01	0.15	
	Positives only	11.1	0.71	
Stevens et al.	Augmented	13.5	0.83	**47%**
	Fully labeled	10.8	1.0	
	Positives only	15.9	1.2	
Perera et al.	Augmented	10.6	0.27	**33%**
	Fully labeled	7.20	0.14	
	Positives only	12.2	0.24	
Neves et al.	Augmented	12.3	2.4	**75%**
	Fully labeled	7.05	0.19	
	Positives-only	27.9	1.4	

5.7. Comparison of Predictions in Regression Models

To compare the performances of the regression models within the yield prediction task, we created Scatter plots of the predictions on the hold-out test set for the Positives only, PAYN augmented, and Fully labeled models (see Figures S10-S13).

For all datasets, the Positive only models A) show a systematic error in the negative data / low yielding area by rarely predicting yields below 20%. This confirms, that lacking negative data caused by reporting bias leads to overestimated yields and the difficulty of distinguishing failure from success. In contrast, the PAYN augmented model B) successfully corrects this bias. The predictions for low-yielding reactions are visually arranged around zero, allowing for meaningful predictions in the low-yield regime. When compared to the Fully labeled model C), the PAYN model exhibits slightly higher variance in the low-yield regime. This is attributed to an assigned artificial yield of 0% to all identified Reliable Negatives, whereas the Fully labeled model learns from the exact experimental values (e.g., distinguishing between 0% and 15%), which presumably contains subtle structure-yield information.

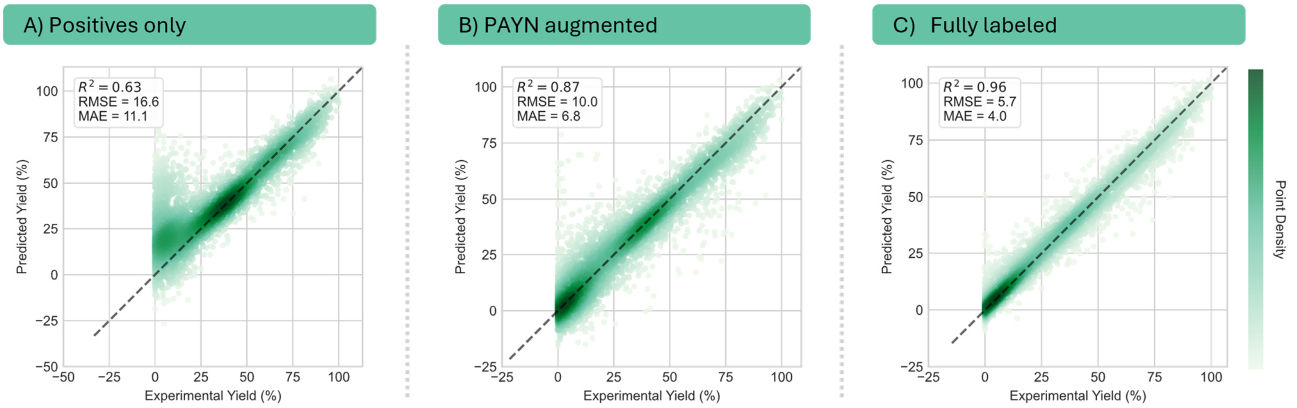

Figure S10: Scatter plots of predicted versus ground-truth yields for the test set of the Ahneman et al. dataset. Models were trained using optimized hyperparameters on A) the Positives Only dataset, B) the PAYN augmented dataset, and C) the Fully labeled dataset (ground truth).

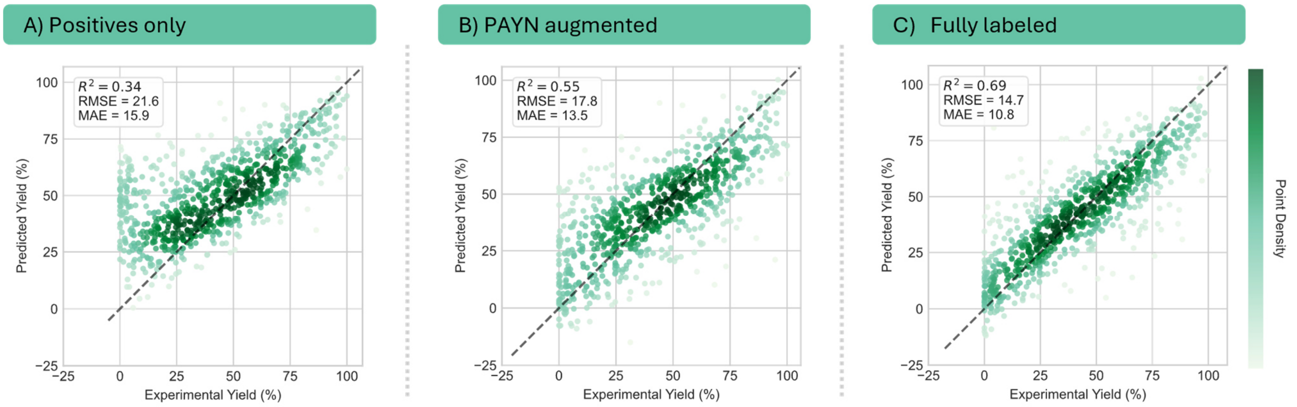

Figure S11: Scatter plots of predicted versus ground-truth yields for the test set of the Stevens et al. dataset. Models were trained using optimized hyperparameters on A) the Positives Only dataset, B) the PAYN augmented dataset, and C) the Fully labeled dataset (ground truth).

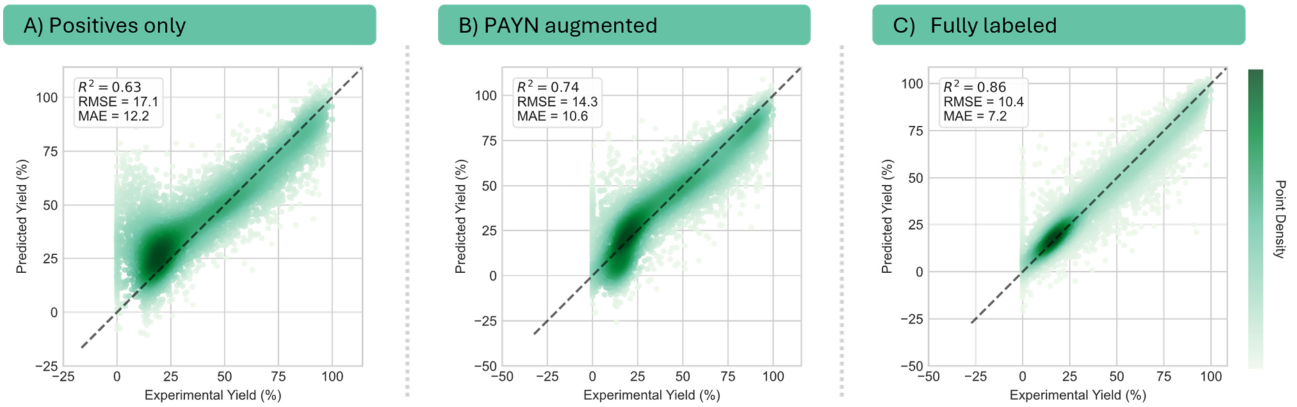

Figure S12: Scatter plots of predicted versus ground-truth yields for the test set of the Perera et al. dataset. Models were trained using optimized hyperparameters on A) the Positives Only dataset, B) the PAYN

augmented dataset, and C) the Fully labeled dataset (ground truth).

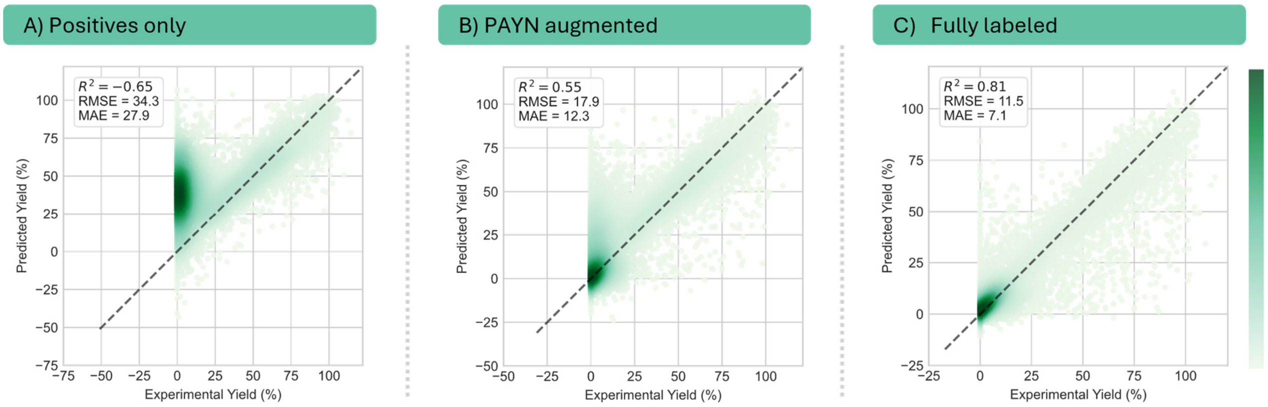

Figure S13: Scatter plots of predicted versus ground-truth yields for the test set of the Neves et al. dataset. Models were trained using optimized hyperparameters on A) the Positives Only dataset, B) the PAYN augmented dataset, and C) the Fully labeled dataset (ground truth).

5.8. Impact of Dataset Size on Performance of the Fully Labeled Model

The fully labeled model was trained using the full training data set. Aside from having access to labeled data, this gives the model the additional advantage of being trained on a much larger training set. To detach the label availability from dataset size, two additional models were trained. These models were both trained on a random subset of the labeled training dataset that matched the size of the augmented and the positives only model, respectively.

The average MAEs over five folds of all models are compared in Table S10. It is worth noting that while size of the fully labeled model and the positives only model stay roughly the same (minor deviations may occur due to splitting), the size of the augmented model, and therefore also its fully labeled counterpart, may vary between folds, depending on the number of datapoints identified as RN.

As expected, reducing the size of the training dataset of the fully labeled models leads to a moderate increase in MAE. This closes the performance gap between the augmented and fully labeled model even further. However, even while having access to the same number of labeled datapoints, the fully labeled models still perform better, which is likely due to two reasons: First, its training data distribution perfectly matches the ground truth. Second, the yield of all RN is assumed to be 0% when training the augmented model. Even when all RN are true negatives (datapoints with a yield below or equal to 20%), this approximation introduces a small systematic error, while the fully labeled model is trained on the correct yield values.

Table S10: Average results of the final models over five folds trained with optimized hyperparameters (see Table S9 for details). Two additional fully labeled models were trained using a subset of the full training set to match the size of the training data of the augmented model (Augmented size) and of the positives only model (PO size). The average size of datapoints in the training set over five folds is given in parentheses. The improvement indicates the improvement in MAE of the augmented model relative to the fully labeled models of varying sizes.

Dataset	Model	MAE (Yield %)	STD (Yield %)	Improvement
Ahneman et al.	Augmented	6.78	0.39	
	Positives only	11.1	0.71	
	Fully labeled (2487)	4.01	0.15	61%
	Fully labeled Augmented size (1381)	5.49	0.34	77%
	Fully labeled PO size (1061)	5.99	0.37	85%
Stevens et al.	Augmented	13.5	0.83	
	Positives only	15.9	1.2	
	Fully labeled (560)	10.8	1.0	47%
	Fully labeled Augmented size (285)	11.8	0.41	59%
	Fully labeled PO size (245)	12.5	1.1	71%

Perera et al.	Augmented	10.6	0.27	
	Positives only	12.2	0.24	
	Fully labeled (4147)	7.20	0.14	33%
	Fully labeled Augmented size (1962)	8.90	0.34	48%
	Fully labeled PO size (1657)	9.37	0.35	57%
Neves et al.	Augmented	12.3	2.4	
	Positives only	27.9	1.4	
	Fully labeled (8155)	7.05	0.19	75%
	Fully labeled Augmented size (2735)	8.27	0.09	79%
	Fully labeled PO size (1809)	8.86	0.30	82%

5.9. Statistical Analysis of the Augmented Model

While there is a clear difference between the average MAEs of the augmented and positive only models (see Table S9), the standard deviations are often quite large, especially for the Stevens et al. dataset. These fluctuations are likely due to variations in the training sets across the folds. To ensure that the augmented model is statistically significantly better than the positive only model, a Wilcoxon signed-rank test was performed. For each dataset, 25 pairs of augmented and positive-only models were trained (each pair was trained and evaluated on the same data). The performances of the 25 models are shown in Table S12. The Wilcoxon signed-rank test demonstrates that the augmented models trained in this work significantly outperform the positive only models (see Table S11).

Table S11: Results of the Wilcoxon signed-rank test between the augmented model and the positive only model as the lower benchmark. 25 paired models were trained over five runs (with different random seeds) over five folds each.

Dataset	Augmented Model MAE (Yield %)	Positives only Model MAE (Yield %)	*W*	*p*
Ahneman et al.	6.72±0.44	10.9±0.52	0	<0.001
Stevens et al.	14.0±1.2	15.8±1.1	14	<0.001
Perera et al.	10.5±0.26	12.4±0.36	0	<0.001
Neves et al.	12.2±2.3	28.1±0.79	0	<0.001

Table S12: Results of augmented and positive only models trained on four different datasets with optimized hyperparameters (see Table S9 for details). For each dataset 25 models were trained, using five different random seeds to initialize each run and performing five-fold cross validation during each run.

Random Seed	Fold	Ahneman et al.		Stevens et al.	
		Augmented Model	Positives only Model	Augmented Model	Positives only Model
42	0	6.38	10.3	13.2	15.8
42	1	6.72	11.2	13.2	15.7
42	2	6.77	11.2	14.9	16.5
42	3	6.61	10.6	12.7	14.2
42	4	7.43	12.1	13.5	17.4
43	0	6.12	11.2	12.2	14.3
43	1	7.00	11.1	14.6	17.6
43	2	6.68	11.5	14.9	14.8
43	3	6.36	9.87	14.2	16.8

43	4	6.54	10.6	15.8	15.7
44	0	7.40	11.7	15.8	15.3
44	1	7.42	11.3	13.6	14.8
44	2	7.17	11.3	14.0	14.3
44	3	6.49	11.3	14.6	16.3
44	4	5.94	9.80	14.9	17.5
45	0	6.29	11.0	13.9	15.7
45	1	7.16	10.9	14.6	16.9
45	2	6.35	11.3	13.1	13.3
45	3	6.87	10.6	13.0	15.3
45	4	6.93	10.6	12.4	16.3
46	0	6.65	10.7	13.4	16.1
46	1	6.50	10.7	12.6	15.1
46	2	6.48	10.6	12.9	16.1
46	3	6.28	11.3	16.1	15.9
46	4	7.62	10.8	16.7	16.9

Random Seed	Fold	Perera et al.		Neves et al.	
		Augmented Model	Positives only Model	Augmented Model	Positives only Model
42	0	10.5	12.4	9.88	26.9
42	1	10.4	12.5	14.6	30.3
42	2	10.5	12.0	9.75	28.2
42	3	11.0	12.3	14.4	27.4
42	4	10.4	12.0	12.9	26.9
43	0	11.0	12.8	10.4	29.2
43	1	11.0	12.6	9.98	27.9
43	2	10.5	11.9	12.7	28.3
43	3	10.5	12.1	9.42	28.3
43	4	10.3	12.2	17.1	29.2

44	0	10.1	12.4	13.5	28.2
44	1	10.8	12.8	16.7	28.3
44	2	10.1	12.9	11.0	27.8
44	3	10.6	12.6	9.88	27.1
44	4	10.7	11.9	10.2	28.2
45	0	10.9	12.6	13.5	28.7
45	1	10.4	13.0	15.2	28.5
45	2	10.3	11.6	11.1	28.2
45	3	10.6	12.6	11.7	28.6
45	4	10.5	12.5	15.4	28.0
46	0	10.4	12.4	12.1	27.4
46	1	10.1	12.9	10.4	27.2
46	2	10.5	12.6	10.1	27.2
46	3	10.5	12.7	13.2	27.2
46	4	10.7	12.0	10.3	28.3

6. REFERENCES

(1) Ahneman, D. T.; Estrada, J. G.; Lin, S.; Dreher, S. D.; Doyle, A. G. Predicting Reaction Performance in C–N Cross-Coupling Using Machine Learning. *Science* **2018**, *360* (6385), 186–190. https://doi.org/10.1126/science.aar5169.
(2) Stevens, J. M.; Li, J.; Simmons, E. M.; Wisniewski, S. R.; DiSomma, S.; Fraunhoffer, K. J.; Geng, P.; Hao, B.; Jackson, E. W. Advancing Base Metal Catalysis through Data Science: Insight and Predictive Models for Ni-Catalyzed Borylation through Supervised Machine Learning. *Organometallics* **2022**, *41* (14), 1847–1864. https://doi.org/10.1021/acs.organomet.2c00089.
(3) Perera, D.; Tucker, J. W.; Brahmbhatt, S.; Helal, C. J.; Chong, A.; Farrell, W.; Richardson, P.; Sach, N. W. A Platform for Automated Nanomole-Scale Reaction Screening and Micromole-Scale Synthesis in Flow. *Science* **2018**, *359* (6374), 429–434. https://doi.org/10.1126/science.aap9112.
(4) Neves, P.; Hao, B.; Aikonen, S.; Diccianni, J. B.; Wegner, J. K.; Schwaller, P.; Strambeanu, I. I. Robust Out-of-Distribution Prediction of Buchwald-Hartwig Reactions. ChemRxiv October 13, 2025. https://doi.org/10.26434/chemrxiv-2025-xcr46.
(5) *Reaxys*. Reaxys. https://www.reaxys.com/ (accessed 2025-07-21).
(6) Strieth-Kalthoff, F.; Sandfort, F.; Kühnemund, M.; Schäfer, F. R.; Kuchen, H.; Glorius, F. Machine Learning for Chemical Reactivity: The Importance of Failed Experiments. *Angew. Chem., Int. Ed.* **2022**, *61* (29), e202204647. https://doi.org/10.1002/anie.202204647.
(7) Gao, W.; Raghavan, P.; Shprints, R.; Coley, C. W. Revealing the Relationship between Publication Bias and Chemical Reactivity with Contrastive Learning. *J. Am. Chem. Soc.* **2025**, *147* (10), 8959–8968. https://doi.org/10.1021/jacs.5c01120.
(8) The pandas development team. Pandas-Dev/Pandas: Pandas, 2025. https://doi.org/10.5281/ZENODO.3509134.
(9) *RDKit*. https://www.rdkit.org/ (accessed 2025-12-18).
(10) Dorogush, A. V.; Ershov, V.; Gulin, A. CatBoost: Gradient Boosting with Categorical Features Support.

arXiv October 24, 2018. https://doi.org/10.48550/arXiv.1810.11363.
(11) Prokhorenkova, L.; Gusev, G.; Vorobev, A.; Dorogush, A. V.; Gulin, A. CatBoost: Unbiased Boosting with Categorical Features. arXiv January 20, 2019. https://doi.org/10.48550/arXiv.1706.09516.
(12) Akiba, T.; Sano, S.; Yanase, T.; Ohta, T.; Koyama, M. Optuna: A Next-Generation Hyperparameter Optimization Framework. In *Proceedings of the 25th ACM SIGKDD International Conference on Knowledge Discovery & Data Mining*; KDD '19; Association for Computing Machinery: New York, NY, USA, 2019; pp 2623–2631. https://doi.org/10.1145/3292500.3330701.
(13) Pedregosa, F.; Varoquaux, G.; Gramfort, A.; Michel, V.; Thirion, B.; Grisel, O.; Blondel, M.; Prettenhofer, P.; Weiss, R.; Dubourg, V.; Vanderplas, J.; Passos, A.; Cournapeau, D.; Brucher, M.; Perrot, M.; Duchesnay, É. Scikit-Learn: Machine Learning in Python. *J. Mach. Learn. Res.* **2011**, *12* (85), 2825–2830.
(14) Harris, C. R.; Millman, K. J.; Van Der Walt, S. J.; Gommers, R.; Virtanen, P.; Cournapeau, D.; Wieser, E.; Taylor, J.; Berg, S.; Smith, N. J.; Kern, R.; Picus, M.; Hoyer, S.; Van Kerkwijk, M. H.; Brett, M.; Haldane, A.; Del Río, J. F.; Wiebe, M.; Peterson, P.; Gérard-Marchant, P.; Sheppard, K.; Reddy, T.; Weckesser, W.; Abbasi, H.; Gohlke, C.; Oliphant, T. E. Array Programming with NumPy. *Nature* **2020**, *585* (7825), 357–362. https://doi.org/10.1038/s41586-020-2649-2.
(15) Sandfort, F.; Strieth-Kalthoff, F.; Kühnemund, M.; Beecks, C.; Glorius, F. A Structure-Based Platform for Predicting Chemical Reactivity. *Chem* **2020**, *6* (6), 1379–1390. https://doi.org/10.1016/j.chempr.2020.02.017.
